# Designing Michaelases: exploration of novel protein scaffolds for iminium biocatalysis†

**DOI:** 10.1039/d4fd00057a

**Published:** 2024-03-18

**Authors:** Alejandro Gran-Scheuch, Stefanie Hanreich, Iris Keizer, Jaap W. Harteveld, Eelco Ruijter, Ivana Drienovská

**Affiliations:** a Department of Chemistry and Pharmaceutical Sciences, Faculty of Science, Vrije Universiteit Amsterdam De Boelelaan 1108 1081 HZ Amsterdam The Netherlands i.drienovska@vu.nl

## Abstract

Biocatalysis is becoming a powerful and sustainable alternative for asymmetric catalysis. However, enzymes are often restricted to metabolic and less complex reactivities. This can be addressed by protein engineering, such as incorporating new-to-nature functional groups into proteins through the so-called expansion of the genetic code to produce artificial enzymes. Selecting a suitable protein scaffold is a challenging task that plays a key role in designing artificial enzymes. In this work, we explored different protein scaffolds for an abiological model of iminium-ion catalysis, Michael addition of nitromethane into *E*-cinnamaldehyde. We studied scaffolds looking for open hydrophobic pockets and enzymes with described binding sites for the targeted substrate. The proteins were expressed and variants harboring functional amine groups – lysine, *p*-aminophenylalanine, or *N*^6^-(d-prolyl)-l-lysine – were analyzed for the model reaction. Among the newly identified scaffolds, a thermophilic ene-reductase from *Thermoanaerobacter pseudethanolicus* was shown to be the most promising biomolecular scaffold for this reaction.

## Abstract 2

The study identified promising protein scaffolds for artificial enzyme development in iminium-ion catalysis, demonstrating activity and enantioselectivity in abiological Michael addition reactions.

## Graphical abstract

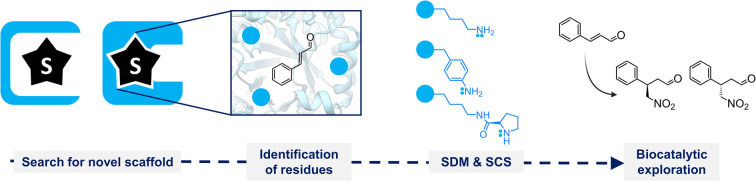

## Introduction

Biocatalysis is becoming a powerful alternative for synthesizing (asymmetric) synthons and valuable molecules for agrochemical, pharmaceutical, and chemical manufacturing industries.^1,2^ Biocatalysts offer tunability and exquisite precision control over the chemo-, regio-, and enantioselectivity of reactions, with high turnover numbers (TONs).^3,4^ However, enzymes are often limited to metabolic reactivities, and unless engineered, traditional synthetic chemical methods exhibit a wider and more versatile range of available reactivities. Nevertheless, this limitation can be addressed by expanding the genetic code by incorporating new-to-nature functional groups.^5–7^ This approach enables the modification of proteins with single-atom precision, enabling the execution of abiological reactivities.^8–10^ Besides the functional group to be incorporated, selecting a suitable biomolecular scaffold is a challenging task that plays a key role in designing artificial enzymes for new-to-nature reactivities. Moreover, appropriate scaffolds are a fundamental starting point for the success of protein-engineering campaigns, and their identification can be tackled in different ways. This selection includes, among others, computational approaches – such as the *de novo* design of enzymes and deep learning – or the direct incorporation of non-canonical amino acids (ncAAs) into natural proteins.^11,12^ For the latter, proteins with or without a predefined active site can be employed, for so-called top-down or bottom-up strategies. In the top-down strategy, enzymes displaying a predefined active site suitable for a desired substrate can offer an attractive starting point.^13–16^ Enzymes can exhibit some initial activity toward the desired reactivity, making them suitable candidates for directed-evolution engineering campaigns. However, their evolution may be constrained by a more restricted protein environment. Conversely, the bottom-up strategy, employing proteins lacking a fixed active site, offers the flexibility to introduce ncAAs at various positions. This approach facilitates the development of versatile artificial enzymes capable of catalyzing multiple reactions, overcoming the substrate specificity observed in natural enzymes.^8,17–20^ Nonetheless, initial activities may be low or even absent, as a comprehensive understanding is critical to determine the optimal positioning of the ncAA.

To date, it is not clear what methodology is superior, and the pursuit of an optimal universal scaffold appears unrealistic. A well-described biomolecular scaffold is LmrR, a lactococcal multidrug-resistance regulator that has been described for the design of several artificial enzymes.^9,18,21–24^ The promiscuous nature of LmrR is attributed to its open hydrophobic pocket, formed from dimeric interactions at the interface of the monomers.^25^ The hydrophobic pocket facilitates the insertion of several ncAAs and diverse substrates. Inspired by organocatalysis, where secondary amines have been predominant in the field because they tend to be better nucleophiles, we recently reported the *in vivo* incorporation of new-to-nature ncAAs harboring functional active secondary amines into LmrR.^24^ LmrR mutants were designed to incorporate stereoisomers of *N*^6^-(prolyl)-l-lysine (d/lProK) and *N*^6^-((*R*)-piperidine-2-carbonyl)-l-lysine. These variants were used to catalyze the Michael addition of nitromethane to *E*-cinnamaldehyde. This reaction is industrially appealing due to its asymmetric carbon–carbon bond-forming nature and for the synthesis of valuable enantiopure γ-nitroaldehydes.^26,27^ The variant incorporating dProK displayed the most promising catalytic profile for iminium ion-based biocatalysis. Nevertheless, further optimization is required for its practical application as a biocatalytic alternative.

**Fig. 1:**
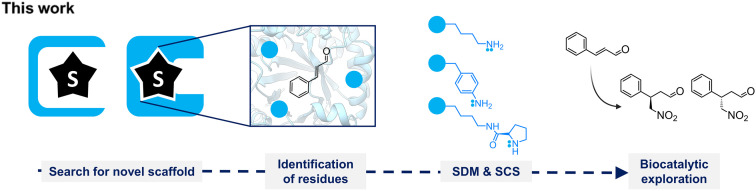
Workflow of this work. Several protein scaffolds were searched for by their hydrophobic pocket or substrate binding pocket for *E*-cinnamaldehyde. Relevant residues were selected as hotspots and mutated to lysine, *p*-aminophenylalanine, or *N*^6^-d-prolyl-l-lysine. Obtained variants were tested as catalysts toward the iminium-ion reaction of Michael addition of nitromethane to *E*-cinnamaldehyde. SDM: site-directed mutagenesis, SCS: stop-codon suppression.

Remarkably, iminium-ion biocatalysis is rare in nature, although a few cases have been described in the literature.^22,23,28–31^ Several examples involve enzymes with primary amines in their active sites, such as the non-canonical amino acid *para*-aminophenylalanine (*p*AF)^8,9,22^ or lysine.^32^ For the latter, the evolution of a class I aldolase, obtaining a 12-fold mutant (DERA-MA) capable of synthesizing (*R*)-4-nitro-3-phenylbutanal, has been reported, where lysine appears to be functionally active due to a reduced p*K*_a_ attributed to the protein architecture of DERA-MA.^33^ In this work, we aimed to explore suitable biomolecular scaffolds for the desired Michael addition reactivity (Fig. 1). We conducted (i) a pocket-guided search to identify proteins with open pockets and (ii) a substrate-guided search to find enzymes known to bind the targeted substrate. Subsequently, we identified relevant positions for incorporating functional groups for iminium catalysis. For this, we evaluated the functional secondary amine dProK, and the primary amines lysine and *p*AF. After protein expression and purification, small-scale conversions using these variants were tested to evaluate the Michael addition of nitromethane to *E*-cinnamaldehyde.

## Results and discussion

The selection of a suitable protein scaffold is crucial in designing artificial enzymes. The identification of biomolecular scaffolds for incorporating ncAAs was approached using two independent methods: (i) a genome mining search for thermophilic homologs of LmrR and QacR. These templates were chosen because they have been described to possess a hydrophobic pocket suitable for ncAA incorporation.^17–19,22^ Both proteins exhibit broad pockets, approximately 1500 Å^3^ in size, and have been studied for the design of metallo- and artificial enzymes. Additionally, (ii) a search was conducted for enzyme structures known to bind chemically similar substrates to *E*-cinnamaldehyde.

For the pocket-guided approach, a genome mining analysis was performed, focusing on genomes of thermophilic organisms and metagenomes from samples isolated from high-temperature sites (Fig. S1†). Initially, a cutoff between 30% and 90% amino-acid sequence identity was applied for LmrR and 15–90% for QacR. To increase diversity, sequences with over 90% redundancy were discarded. After applying these constraints, 102 sequences for LmrR-like proteins and 15 for QacR-like proteins were selected. The final selection was made considering cladogram analysis for sample diversity, homology models, and the CAVER tool for visualizing tunnels and channels in protein structures.^34^ From this analysis, five putative scaffolds were selected, designated as BbmrR, CdmrR, TbmrR, AtRegR, and TmRegR (Table 1). The scaffolds BbmrR, CdmrR, and TbmrR have a sequence identity of 40–45% with LmrR, while AtRegR and TmRegR have 18% and 17% sequence identity with QacR, respectively.

**Table 1:** Selected scaffolds. Biomolecular scaffolds were chosen by a pocket- and a substrate-guided search. Further selection was done depending on their expression and stability

Putative scaffold	NCBI protein code	Source	Selection	Expression	*T*_i_ a [°C]
BbmrR	WP_171505753	*Brevibacillus borstelensis*	Pocket-guided	Soluble	75
AtRegR	WP_043966894	*Anoxybacillus thermarum*	Pocket-guided	Partially	79
CdmrR	WP_020156622	*Caldibacillus debilis*	Pocket-guided	Insoluble or low yield	n.d.
TbmrR	HHX23360	*Thermoanaerobacterales bacterium*	Pocket-guided	Insoluble or low yield	n.d.
TmRegR	WP_004080830	*Thermotoga* sp.	Pocket-guided	Insoluble or low yield	n.d.
LmrR	A2RI36	*Lactococcus lactis*	Template	Soluble	66
TOYE	3KRU_A	*Thermoanaerobacter pseudethanolicus* E39	Substrate-guided	Soluble	>80
EncP^b^	AAF81735	*Streptomyces maritimus*	Substrate-guided	Soluble	65
VAO^c^	P56216	*Penicillium simplicissimum*	Substrate-guided	Soluble	56

a *T*_i_, inflection temperature. Inflection temperature of the unfolding transition in the 350 nm/330 nm fluorescence ratio signal.

b EncP_R299K mutant has been described to accept the targeted substrate.^36^

c VAO_H61T was selected to obtain a larger pocket by removing the covalently bound FAD.^38^

For the substrate-guided approach, the enzymes TOYE (a thermophilic ene-reductase isolated from *Thermoanaerobacter pseudethanolicus* E39), EncP_R299K (a bacterial ammonia-lyase mutant from *Streptomyces maritimus*), and VAO (a flavin-dependent vanillyl-alcohol oxidase from *Penicillium simplicissimum*) were chosen; these proteins exhibit relatively high thermostability and purification yields (Table 1).^35–37^ For VAO, aiming for a larger inner pocket, the mutant H61T was chosen because it has been described as a critical residue in the covalent flavination of this oxidase.^38^ This mutant was shown to be expressed almost completely as the apoprotein, exhibiting an empty FAD-binding pocket.

Among the eight chosen scaffolds, all were predicted as soluble, or without transmembrane regions.^39^ The retrieved sequences were codon optimized for expression in *E. coli* and ordered into a pET-21(+) vector containing a C-terminal 6x histidine tag. The constructs were then transformed into *E. coli* BL21(DE3) cells. TOYE, EncP_R299K, VAO_H61T, BbmrR, and AtRegR showed decent expression levels, confirmed by SDS-PAGE purity (Fig. S2†) and LC-MS analysis (Fig. S3†). Variants CdmrR, TbmrR, and TmRegR were quickly discarded, as it was not possible to obtain soluble proteins under any tested conditions, including variations in temperature, expression time, and inducer concentration. For AtRegR, purification conditions were further adjusted by using a higher ionic strength in the purification buffers (1 M NaCl) due to its tendency to precipitate. However, no significant improvement was observed. Consequently, due to this solubility issue, AtRegR was not further investigated. Since BbmrR was identified as an LmrR-like protein, its predicted oligomeric state is expected to exhibit a similar dimeric interface, allowing for the formation of an open pocket.^25^ BbmrR was observed as a dimer *via* size-exclusion chromatography (Fig. S4†). Interestingly, BbmrR proved to be highly thermostable, with an inflection temperature (*T*_i_) of 75 °C, 10 °C higher than that of LmrR. Additionally, in the presence of 10% v/v ethanol as a cosolvent, while the *T*_i_ of LmrR decreased from 65 to 53 °C, BbmrR exhibited a decrease of only 8 °C.

**Fig. 2:**
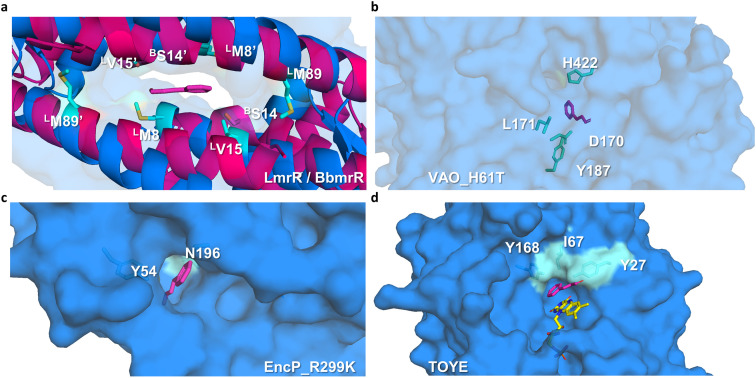
Selection of residues for mutagenesis. (a) For BbmrR (pink ribbon), analysis was carried out by comparing it with LmrR (blue ribbon), as a previously described active scaffold. Positions S14 of BbmrR are highlighted along with the residues chosen for LmrR, labelled with B or L, respectively. (b–d) Substrate-guided scaffold surfaces of (b) VAO_H61T, (c) EncP_R299K, and (d) TOYE are shown in light blue. The transparency settings for VAO_H61T are adjusted to highlight the inner residues. The substrate is shown in pink (PDB 9Y6), and selected residues are highlighted in cyan. Modeling was performed using the program AutoDock (https://vina.scripps.edu/).

Preliminary docking analysis of *E*-cinnamaldehyde with the substrate-guided selected proteins was conducted to identify relevant residues for the incorporation of functional amines (Fig. 2). For BbmrR, structure analysis and a multiple sequence alignment (MSA) with LmrR suggested position S14 as relevant for the design (Fig. S5†). This position is structurally homologous to residue V15 in LmrR, which has previously been identified as a hotspot for testing initial activity when employing LmrR as an artificial enzyme.^18,22,24^ Additionally, LmrR positions M8 and M89 were also included in this analysis, as previous studies have shown their importance in the design of artificial enzymes.^18,40^ For the proteins identified through the substrate-guided approach, the following positions based on docking predictions and prior knowledge of the active site were chosen: TOYE Y27, I67 and Y168; EncP_R299K Y54 and N196; and VAO_H61T D170, L171, Y187 and H422.

The low availability and high cost of some ncAAs pose significant challenges for the utilization of artificial enzymes. Additionally, weak interactions with their respective aminoacyl-tRNA synthetase and low incorporation into proteins are problems that can be addressed through protein-engineering campaigns.^41–43^ For dProK, similar to other pyrrolysine systems,^44^ this issue has not yet been addressed. Therefore, after selecting the relevant positions, we started by evaluating the selected targets and their primary amine variants. As a result, the number of proteins containing the dProK ncAA to be tested was reduced, avoiding residues that could be critical for expression, folding, and/or solubility. Site-directed mutagenesis was performed for all scaffolds using the QuikChange methodology (oligo DNAs listed in Table S1†). Proteins were then expressed and purified (Fig. S6†), and confirmed by LC-MS (Fig. S7–S11†). For the incorporation of *p*AF, the orthogonal system utilized *p*-azidophenylalanine (*p*AzF);^45^ hence, variants were obtained after mild incubation with TCEP (tris(2-carboxyethyl)phosphine) as a Staudinger reducing agent (Scheme S1†). The reduction of the ncAA was confirmed by LC-MS (Fig. S7–S11†). Additionally, the thermostability of all variants was assessed, with only minor variations observed (<2 °C).

**Fig. 3:**
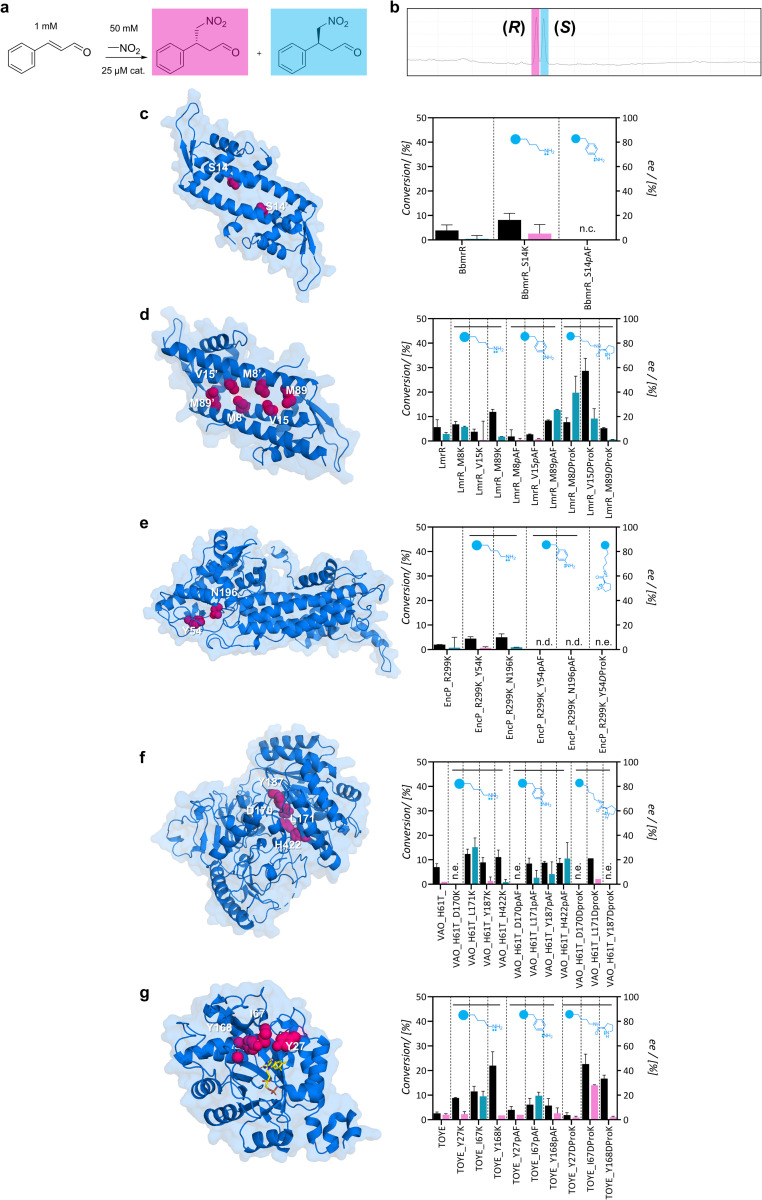
Michael addition of nitromethane to *E*-cinnamaldehyde using the scaffolds as catalysts. (a) Small-scale conversions were formulated in 50 mM HEPES, 150 mM NaCl and 5% v/v EtOH at pH 6.5; (b) enantiomeric excess was calculated from chromatograms – the (*R*)-product is shown in pink and the (*S*)-product in blue; (c–g) conversion levels and ee of the desired reaction using variants of BbmrR, LmrR, EncP_R299K, VAO_H61T or TOYE as catalysts. Residues evaluated during this study are highlighted in pink in the structures. n.c. indicates no conversion, n.d. indicates not determined (due to precipitation of samples), and n.e. indicates no expression.

Subsequently, small-scale conversions were optimized by evaluating the effect of pH in the uncatalyzed reaction, and the catalytic effect of 25 μM pyrrolidine or aniline – functional groups at the side chains of dProK and *p*AF (Fig. S12†). While the uncatalyzed reaction was favoured at higher pH, it resulted in a racemic mixture of 4-nitro-3-phenylbutanal. Further reactions were formulated at pH 6.5 to suppress the uncatalyzed reaction and were monitored by gas chromatography (Fig. 3a and b). Control reactions with aniline and pyrrolidine exhibited minor conversions after 24 hours (5 ± 2% and <5% ee). For entries using wild-type variants or initial scaffolds, conversions were low (3–8%) and did not show clear enantioselectivity (Fig. 3). The lack of activity for the majority of the scaffolds is not surprising. Although they have space to accommodate the substrate, they do not possess the functional groups necessary to increase electrophilicity and activate the carbonyl group for nucleophilic attack. Other factors, such as the entropic effect, also play a relevant role when there is some activity despite a lack of functional groups, by increasing the interaction between the substrates. For these cases, a racemic product can be expected.

Conversely, some lysine variants displayed higher Michael addition activity than wild-type scaffolds after 24 hours. Moreover, although modest, some enantioselectivity was observed in the reaction, suggesting that protein architecture influences the enantioselectivity of the reaction. Despite extending the reaction time up to 48 hours, conversion levels did not significantly change. In particular, TOYE_I67K, TOYE_Y168K, and VAO_H61T_L171K displayed moderate conversion. In contrast, BbmrR did not show promising results as a scaffold; while BbmrR_S14K exhibited slightly higher conversion levels than the uncatalyzed reaction, it remained inactive when incorporating *p*AF (Fig. 3c). This may be attributed to a ‘closed’ hydrophobic pocket, although further studies are required to confirm this. Consequently, BbmrR variants were discarded for further investigation.

For the LmrR variants, all three positions with the primary amines (M8, V15 and M89) showed poor conversion levels; LmrR_M8K displayed a slight preference for the (*S*)-nitroaldehyde (12% ee) (Fig. 3d). When incorporating *p*AF, the product yield was lower for all three variants. Interestingly, LmrR_M89*p*AF exhibited 25% ee towards the (*S*)-product, albeit with a moderately poor conversion rate of 8%. Unfortunately, EncP_R299K lysine variants exhibited poor activity towards *E*-cinnamaldehyde without clear enantioselectivity (4–5% conversion), and the *p*AF variants tended to precipitate. Only position Y54 was later examined for the incorporation of dProK. In the case of VAO_H61T, all lysine and *p*AF variants exhibited higher conversions than VAO_H61T, except for mutants at position D170, which were poorly expressed (Fig. 3f). Position D170 was further investigated because it is a relevant site in VAO, acting as an active residue.^46^ Position L171K showed 14% conversion with a preference for the (*S*)-product (30% ee), whereas the *p*AF variant produced a racemic mixture. Position H422 exhibited moderate initial conversion levels (10–14%), and a preference up to 20% ee towards the synthesis of the (*S*)-product. Finally, for the TOYE variants, TOYE_I67K displayed a slight enantioselective preference for the (*S*)-product (19% ee) and Y168K gave racemic products (Fig. 3g). Conversion levels were 11% and 25%, respectively. I67*p*AF and Y168*p*AF exhibited decreased conversion with a similar enantioselective profile compared with the lysine variants (19% ee towards the (*S*)-product and racemic, respectively). TOYE_Y27 variants did not show attractive catalytic properties.

Overall, lysine variants showed slightly improved reactivity compared to *p*AF, possibly due to reasons such as the increased flexibility of lysine for the protein architecture. Conversely, for *p*AF, the resonance effect with the adjacent π system reduces the availability of the lone pair on the nitrogen atom in the aniline moiety, potentially resulting in lower nucleophilicity.^47^ The following variants were chosen for the incorporation of dProK: LmrR_M8, V15, and M89; TOYE_Y27, I67, and Y168; EncP_R299K_Y54; and VAO_H61T_D170, L171, and Y187. These variants were selected based on their activity with primary amine variants, catalytic preference for enantiomers, and/or solubility and expression (Fig. S13†). To prevent the cleavage of dProK during protein expression, cultures were supplemented with 5 mM nicotinamide as a CobB inhibitor.^48^ The proteins exhibited the expected mass corresponding to the incorporation of the intact ncAA (Fig. S14†). Small-scale conversions were carried out with 25 μM (2.5% loading) of artificial enzyme as a catalyst for 24 hours. LmrR variants V15dProK and M8dProK showed a preference towards the (*S*)-product with conversions of 28% and 9%, respectively, and ee of 18 and 40%. Reactions using the LmrR_M89dProK variant as a catalyst showed similar conversion levels to the control. Unfortunately, EncP_R299K_Y54dProK could not be purified as a soluble protein. When incorporating BocK at the same position as a positive control for the aminoacyl-tRNA synthetase, the protein was obtained and confirmed by LC-MS, but it still exhibited a tendency to precipitate after freezing. This evidence, along with observations during the incorporation of primary amines, suggests that this residue might be critical for its stability or folding. Among the VAO variants, only L171dProK was obtained as a soluble protein and showed a similar catalytic profile to LmrR_M89dProK. For the TOYE variants, incorporation at positions I67 and Y168 resulted in the highest conversions, 23 and 16%, respectively. The incorporation of the functional secondary amine at position Y168 maintained the racemic production of 4-nitro-3-phenylbutanal, similarly to its lysine variant. However, for TOYE_I67dProK, the artificial enzyme changed the enantioselectivity from 19% towards the (*S*)- to 30% towards the (*R*)-product. Moreover, both TOYE variants maintained thermostability comparable to that of the wild-type. Reactions using both TOYE variants were tested with double the amount of nitromethane as the nucleophile, yet the conversion and enantioselectivity profiles remained similar (Fig. S15†).

## Conclusions

In this study, we conducted a search to identify biomolecular scaffolds suitable for use as artificial enzymes in an iminium-ion model reaction – specifically, the Michael addition to *E*-cinnamaldehyde. One approach was focused on identifying scaffolds with an open pocket, facilitating further ‘bottom-up’ engineering. However, none of the newly ‘pocket-guided’ identified scaffolds proved appealing for further investigation, either due to issues with solubility or inactivity. Conversely, the previously described multidrug-resistance regulator LmrR, when incorporating dProK, exhibited moderate activity and a slight preference for the (*S*)-product. Variants harboring the pyrrolidine-ncAA at positions M8 and V15 were shown to be the most appealing scaffolds for further optimization. Interestingly, the evolution of DERA-MA for the same reaction has been described with ee = 99% for the (*R*)-product; however, variants able to access the (*S*)-product were not obtained. The selection of either of these artificial enzymes for an enzyme-engineering campaign could unlock the optically pure synthesis of the desired product. Simultaneously, we explored enzymes already possessing a binding pocket for the target substrate, employing a substrate-guided approach for ‘top-down’ engineering. Some scaffolds showed activity, with TOYE_I67K, TOYE_Y168K, VAO_H61T_L171K, TOYE_I67dProK, and TOYE_Y168dProK appearing to be the most promising proteins. Particularly, I67dProK demonstrated higher conversion levels and a preference of 30% ee towards the (*R*)-product. Overall, the proteins selected in this study seem to have high evolvability, with even a single-point mutation showing activity for the abiological reactivity, with TONs up to 12 and TOF (turnover frequency) of 0.5 h^−1^. Additionally, they exhibited relatively high thermostability, with *T*_i_ ranging from 55 to 70 °C. A similar conversion profile was observed during the directed evolution of DERA-MA towards the same reaction, in which the first round exhibited a TON of 42 and TOF of 1.4 h^−1^.^32^ Both the selection of lysine and *N*^6^-prolyl-l-lysine appear highly attractive for a directed-evolution campaign. Biochemically, lysine is appealing due to its easy incorporation as a canonical amino acid. Conversely, although dProK shows lower incorporation levels, its initial design was inspired by organocatalysis, where secondary amines have dominated the field due to their superior behaviour as nucleophiles. Therefore, evolving scaffolds harbouring dProK using a robust incorporation platform is extremely attractive for biotechnological applications.

## Experimental

### General methods

Commercially available reagents were purchased from Sigma-Aldrich, Fischer, Iris or Fluorochem and were directly used without additional purification. Solvents were purchased from VWR Chemicals, Biosolve B.V. or Sigma-Aldrich and used without purification. Bacterial growth media were purchased from Roth. Plasmid pEVOL-pAzF was a gift from Prof. Peter Schultz (Addgene plasmid #31186). Plasmid pEVOL-ABK was a gift from Andrea Musacchio (Addgene plasmid #126035).

### Synthetic procedures

The synthetic procedure for synthesizing *N*^6^-prolyl-l-lysine was carried out as previously described by our group, with minor modifications (Scheme S2†).^24,49^ The synthesis of Cbz-l-Lys-OMe was carried out from Cbz-l-Lys-OH, and after its esterification, the procedure was continued as previously described. The final product was confirmed by NMR, LC-MS and polarimetry (Fig. S16†). NMR spectra were recorded on a Bruker Avance 600, Bruker Avance 500 or Bruker Avance 300, using CDCl_3_, CD_3_OD or DMSO-d_6_ as solvents. The spectra were calibrated using the residual CHCl_3_, CH_3_OH or DMSO as internal standards. Chemical shifts (*δ*) are reported in ppm and coupling constants in Hz. The signals are described as s (singlet), d (doublet), t (triplet), q (quartet), bs (broad singlet) and m (multiplet), or their combinations. Specific optical rotations were recorded on an automatic P3000 polarimeter (Krüss), *λ* = 589 nm, at 1 g 100 mL^−1^.

### Identification of suitable protein scaffolds

Protein scaffolds crystallized with *E*-cinnamaldehyde were identified using the Protein Data Bank, code 9Y6. Thermophilic scaffolds were identified using the Lactococcal multidrug-resistance regulator LmrR (UniProt code A2RI36) and *Staphylococcus aureus* HTH-type transcriptional regulator QacR (UniProt code P0A0N4) sequences as templates for sequence-driven genome mining, using the NCBI server for BLAST searches limited to thermophilic organisms and thermophilic metagenomes. After initial screening, 170 sequences were obtained by selecting targets with an amino-acid sequence identity of between 30–90% with LmrR and QacR. Subsequently, redundancy within the targets was reduced by using the CD-HIT package.^50^ Sequences of the putative scaffolds were clustered and filtered using a sequence identity cutoff of 90%. Multiple sequence alignments and phylogenetic analyses were performed for the resulting 102 sequences for LmrR and 15 for QacR. Both data analyses were performed using Geneious Prime® 2021.2.2. The cladogram was reconstructed using a Neighbor Joining algorithm and the Jukes-Cantor model was used for distance measures (500 bootstrap replications). For LmrR-like scaffolds, PadR1 and PadR2 proteins were used as negative samples. Selected proteins were modelled using AlphaFold, and variants that showed a closed pocket were discarded. Prediction of the transmembrane regions was carried out using the online tool DeepTMHMM.^39^ Docking analyses were modelled using AutoDock Vina and the 9Y6 PDB structure for the ligand.^51^ X-ray structures or AlphaFold models were used for the analysis;^52^ water and external ligand molecules were removed, and hydrogens and ‘Kollman’ charges were added. To prepare a suitable receptor for ligand docking, a simulation cell was defined by amino acids of the active site cavities.

### Molecular biology

*E. coli* strains DH5α and NEB10β were used for cloning and *E. coli* BL21(DE3) was used for expression. DNA sequencing was performed by Eurofins Genomics (Germany). Primers were synthesized by Integrated DNA Technologies (the Netherlands). PfuTurbo DNA polymerase was purchased from Agilent and restriction endonucleases from New England Biolabs (United Kingdom). The plasmid isolation kit and gel extraction kit were purchased from Qiagen (Germany). For the in-house pEVOL MbPylRS construction, two copies of the wild-type PylRS gene were recloned within the pEVOL_AbK plasmid.^24^ One copy of PylRS was cloned using restriction enzymes, and both plasmids were digested with *Nde I* and *Pst I* according to the manufacturer's guidelines. The mixture was incubated at 37 °C overnight. Fragments of interest were then separated on a 1% agarose gel by electrophoresis and the cut DNA fragments were purified using a gel extraction kit. Ligation of both the PylRS insert and pEVOL vector backbone was performed in a 20 μL reaction consisting of 1× T4 DNA reaction buffer, 5 U T4 DNA ligase (Thermo Fisher), and cut insert DNA and vector DNA in a molar ratio of 1 : 2. The mixture was incubated overnight at 16 °C and 8 μL were transferred into chemocompetent *E. coli* DH5α cells. The second copy of the PylRS gene was cloned based on FastCloning.^53^ For both the PylRS gene and pEVOL vector backbone, a 20 μL PCR reaction was prepared with the following components: 1 ng μL^−1^ DNA template, 0.2 mM dNTPs, 0.3 μM forward and reverse primer, 1× PfuTurbo buffer, and 2 U PfuTurbo polymerase. Primers for vector backbone amplification are as follows: (5′), Fw: ATA AGT CGA CCA TCA TCA TC and Rv: GGA TCT AAT TCC TCC TGT TAG. Primers for insert amplification are as follows: (5′), Fw: TAA CAG GAG GAA TTA GAT CCA TGG ATA AAA AAC CGC TGG ATG and Rv: GAT GAT GAT GGT CGA CTT ATT TAC AGG TTC GTG CTA ATG C. The following PCR protocol was used: 94 °C for 30 s; 94 °C for 30 s, 55 °C for 30 s and 72 °C for 2 min (30 cycles); extension at 72 °C for 5 min and hold at 4 °C. The PCR products were digested with restriction endonuclease DpnI for 3 h at 37 °C. After digestion, vector backbone DNA and insert DNA were mixed in a 1 : 4 volume ratio and 5 μL were transformed into chemocompetent *E. coli* DH5α cells.

After identifying protein scaffolds, DNA sequences were codon-optimized for *E. coli* and synthesized by Twist Bioscience (USA). Genes were obtained in pET-21(+) vectors and transformed into chemocompetent *E. coli* BL21(DE3) cells. Mutants were prepared according to QuikChange methodology. Primers were designed using AAscan software (Table S1†).^54^ For the site-directed mutagenesis, 25 μL reactions were prepared as reported by the manufacturer's recommendations (PfuTurbo DNA polymerase, Agilent) with the following PCR program: 95 °C for 2 min; 95 °C for 30 s, 55 °C for 30 s and 72 °C for 7 min (26 cycles); extension at 72 °C for 10 min and hold at 4 °C. PCR products were then digested with restriction endonuclease DpnI at 37 °C for 16 h and 5 μL were transformed into chemocompetent *E. coli* NEB10β cells. Validation of the correct construct was confirmed by sequencing. For ncAA incorporation *via* SCS methodology, plasmids encoding for TAG-scaffold variants were co-transformed with the respective incorporation system (pEVOL *p*AzF^45^ or pEVOL MbPylRS) into chemocompetent *E. coli* BL21(DE3) cells.

### Protein expression and purification

Single colonies of *E. coli* BL21(DE3) cells harboring the respective vectors were chosen for preinoculums overnight at 37 °C. Inoculums were prepared in 1 : 100 dilutions for 50–1000 mL, in lysogeny broth, supplemented with 35 μg mL^−1^ chloramphenicol and 50 μg mL^−1^ ampicillin at 37 °C with constant shaking at 135 rpm. Subsequently, at an optical density (600 nm) of 0.6–0.7, the culture was supplemented with the corresponding ncAAs at 1 or 10 mM, for *p*AzF or dProK, respectively, and induced with 0.05% w/w l-arabinose and 1 mM IPTG at 28 °C for 16 h or 24 h. Additionally, for dProK incorporation, cultures were supplemented with 5 mM nicotinamide as a CobB inhibitor. Cultures were harvested by centrifugation (4000*g* for 20 min at 4 °C) and resuspended in 50 mM TRIS·HCl, 500 mM NaCl, pH 8.0 and 1 mM PMSF. Cell-free extracts (CFEs) were obtained after lysing by sonication (5 s on and 5 s off, for 5 min at 35% amplitude, Branson 550; microtip model 102C (CE)) and then harvesting at 14 000*g* for 10 min at 4 °C. Purified variants were obtained *via* immobilized-metal affinity chromatography using nickel sepharose fast-flow resin (Cytiva). The resin was pre-equilibrated with 50 mM TRIS·HCl, 500 mM NaCl, pH 8.0 (buffer A), then CFEs were loaded into the resin and the mixture was incubated at 4° for 15 min. The resin was washed with 10 CV (column volume) of buffer A with 40 mM imidazole and eluted with buffer A with 400 mM imidazole. For variants harboring a C-terminal strep-tag, CFEs were loaded onto a Step-Tactin column (Strep-Tactin® Superflow® high-capacity resin, IBA Lifesciences) which was prior equilibrated with buffer A. The mixture was incubated for 30 min at 4 °C followed by washing with 10 CV of buffer A. The desired proteins were eluted with buffer A with 2.5 mM desthiobiotin. Elution fractions were loaded on pre-equilibrated Econo-Pac 10DG desalting columns (Bio-Rad), 50 mM HEPES, 150 mM NaCl, pH 6.5. For *p*AzF-variants, *p*AF was obtained by reduction using 1–10 mM TCEP on ice for 2–16 h (except for BbmrR, where 100 mM TCEP was used), and a subsequent buffer exchange. Protein concentration was determined by using the calculated extinction coefficient calculated with Geneious Prime® 2021.2.2 (protein sequences in ESI†). For *p*AF-variants, the absorbance was corrected (*ε*_280_ = 1333 M^−1^ cm^−1^). The final samples were flash-frozen with liquid nitrogen and stored at −70 °C. The purity of each purified batch was confirmed by SDS-PAGE analysis, and successful ncAA incorporation was confirmed by LC-MS.

### Size exclusion chromatography and SDS-PAGE

The oligomeric state of BbmrR was determined *via* analytical size-exclusion chromatography on a Superdex 200 10/300 gel filtration column (Cytiva). The column was pre-equilibrated with 50 mM phosphate buffer, 500 mM NaCl, pH 7.5, and 100 μL of protein sample were injected. For calibration of the column, a series of proteins from the standard gel-filtration calibration LMW/HMW kits (Cytiva) were used. SDS-PAGE samples were prepared by mixing a concentration of 5–20 μg protein with standard Coomassie loading dye and incubated at 95 °C for 10 min. Samples were centrifuged (except for CE) and loaded into a precasted 4–20% gradient gel (GenScript) for 1 h at 135 V and stained using Instant Blue Coomassie (Abcam).

### Mass determination through LC-MS

The majority of the protein analysis was performed on an InfinityLab II LC coupled to a single quadrupole MS (LC/MSD XT, Agilent Technologies) using an AdvanceBio RP-mAb Wide Pore Reversed-Phase column (2.1 × 50 mm, particle size 3.5 μm, Agilent Technologies) and a linear gradient of 0–95% B over 15 min (solvent A: H_2_O + 0.1% formic acid; solvent B: 80% isopropanol, 10% acetonitrile, 10% H_2_O + 0.1% formic acid; flow rate of 0.3 mL min^−1^). The protein samples were analyzed in positive mode as well as by UV absorbance at 210 and 280 nm. Deconvolution was performed with the Agilent OpenLAB CDS ChemStation LC/MS software using standard parameters and 2000 for the noise cut-off. For VAO and TOYE variants, analysis was performed on an Agilent 6230 ESI-TOF using the same column and eluent gradients. The calculated molecular weight of each protein was derived using Geneious Prime® 2021.2.2.

### Thermostability assay

The thermostability of the proteins was determined by measuring the fluorescence response to a temperature ramp (sum brightness at 350 and 330 nm) using a Tycho NT.6 instrument (Tycho, Nanotemper). Protein samples were set at 20 μM following manufacturer recommendations. The capillary was heated from 35 to 95 °C, at a defined rate of 30 °C min^−1^. The inflection temperature was determined as the maximum of the derivative of the sigmoidal curve of the thermogram.

### Small-scale conversion

Small-scale conversions of Michael addition of nitromethane into *E*-cinnamaldehyde were tested. Reactions were formulated at 200–500 μL in 1.5 mL tubes in 50 mM HEPES, 150 mM NaCl and 5% v/v ethanol at pH 6.5. Reactions were prepared with 25 μM protein, 1 mM *E*-cinnamaldehyde and 50 or 100 mM nitromethane, using ethanol as a cosolvent. Reactions were performed in duplicate, except for those of VAO_H61T_dProK variants, which were carried out with *n* = 1 due to low expression and conversion. Controls without protein were carried out in triplicate. Reactions were incubated at 25 °C or 30 °C for 24–48 h with constant gentle shaking. Subsequently, samples were extracted with one volume of ethyl acetate containing 0.02% v/v mesitylene as an external standard for 30 s. To remove residual water, anhydrous magnesium sulfate was added to the organic solution. Identification, separation, and quantification were carried out using a GC-2010 Ultra instrument (Shimadzu) with a Chiraldex GT-A column (0.12 μm × 0.25 mm × 30 m) and helium as the mobile phase. The employed method was 70 °C for 1 min, gradient to 170 °C at 10 °C min^−1^, and hold for 25 min, with a 1 μL injection volume and split ratio of 2. For the identification of 4-phenyl-3-nitrobutanal, the racemic product was synthesized according to a slightly modified reported literature procedure by using pyrrolidine instead of a chiral organocatalyst.^55^ Additionally, 5 μM DERA-MA enzyme was used as a biocatalyst in a 100 mL small-scale reaction, which is described to produce the (*R*)-product with ee = 99%,^32^ formulated with 5 mM *E*-cinnamaldehyde and 50 mM nitromethane, in 50 mM HEPES, 150 mM NaCl and 10% ethanol at pH 6.5. The product was obtained after flash chromatography with a 50% yield (Fig. S17a†). Flash chromatography was performed on Silicycle Silica-P Flash Silica Gel (particle size 40–63 μm, pore diameter 60 Å). Calibration curves were employed to measure conversion levels. For the substrate, *R*^2^ = 0.9994, limit of detection = 347 μM and limit of quantification = 114 μM; for the product, *R*^2^ = 0.9963, LOD = 253 μM and LQD = 83 μM (Fig. S17b†).

## Author contributions

ID conceptualized the study. AG-S, SH, and IK performed the biological experiments. JH performed the chemical synthesis and analysis of the ncAA. ER provided supervision for the synthesis of ncAA. ID provided supervision. AG-S, SH, and ID prepared the manuscript.

## Conflicts of interest

There are no conflicts to declare.

## Supplementary Material

FD-252-D4FD00057A-s001
