# Supplementary material for: Designing Michaelases: exploration of novel protein scaffolds for iminium biocatalysis
Source: Faraday Discuss. 2024 Mar 18;252:279–94. doi: 10.1039/d4fd00057a (PMC11389850; doi:10.1039/d4fd00057a)
Supplement: FD-252-D4FD00057A-s001 [file FD-252-D4FD00057A-s001.pdf]

Electronic Supplementary Material (ESI) for Faraday Discussions.
This journal is © The Royal Society of Chemistry 2024

Supporting Information

Designing Michaelases: Exploration of novel protein scaffolds for iminium biocatalysis

Alejandro Gran-Scheuch,[a] Stefanie Hanreich,[a] Iris Keizer,[a] Jaap Harteveld,[a] Eelco Ruitjer[a] & Ivana Drienovská[a,*]

Biocatalysis is becoming a powerful and sustainable alternative for asymmetric catalysis. However, enzymes are often restricted to metabolic and less complex reactivities. This can be addressed by protein engineering, such as incorporating new-to-nature functional groups into proteins through the so-called expansion of the genetic code to produce artificial enzymes. Selecting a suitable protein scaffold is a challenging task that plays a key role in designing artificial enzymes. In this work, we explored different protein scaffolds for an abiological model of iminium ion catalysis, Michael addition of nitromethane into *E*-cinnamaldehyde. We studied scaffolds looking for open hydrophobic pockets and enzymes with described binding sites for the targeted substrate. The proteins were expressed and variants harboring functional amine groups —lysine, *p*-aminophenylalanine, or N^6-(D-prolyl)-L-lysine— were analyzed toward the model reaction. Among the newly identified scaffolds, a thermophilic ene-reductase from *Thermoanaerobacter pseudethanolicus* exhibited to be the most promising biomolecular scaffold for this reaction.

Table of content

Amino acid sequences of scaffolds 2
Schemes 3
Supporting table 4
Supporting figures 6

[a] *Department of Chemistry and Pharmaceutical Sciences, Faculty of Science, Vrije Universiteit Amsterdam, De Boelelaan 1108, 1081 HZ Amsterdam, The Netherlands. *E-mail: i.drienovska@vu.nl*

Amino Acid Sequences of Selected Scaffolds

LmrR (NCBI A2RI36)
GAEIPKE**M**LRAQTN**V**ILLNVLKQGDNYVYGIIKQVKEASNGEMELNEATLYTIFDRLEQDGIISSYWGDESQGGRRKYYRLTEIGHEN**M**RLAFE
SWSRVDKIIENLEANKKSEAIKSRGGSGGGSHHHHHH*

BbmrR (NCBI WP_171505753)
MDETLNNSDLVRG**S**IDTIILSVLLTGDNYGYQIIKEIYRKSQNRFELKEPTLYSSLRRLEKQKMIESYWGEETQGGRRKYYSITELGRELYRTN
CAEWELARNLIDRLIRVEKDVEERLEHHHHHH*

CdmrR (NCBI WP_020156622)
MNGKISADLLRGHTDTIVLSILMQGDHYGYEIYKTILEKTEGLYELKEATLYSSYKRLEKEGCIIAYWGDETQGGRRKYYRITEKGRQQYYQNK
ADWEFTKKILNKLLGEEQGLEHHHHHH*

TbmrR (NCBI HHX23360)
MGNTISTDLIRGHTDTIILNVLRQGDSYGYEIYKKIIELSGNQYELKEATLYTAFRRLEQDGYVWSYWGDETQGGRRKYYRITDEGKKFYEQSK
HAWDFAKGVLDKLIKGGLDNAKDLEHHHHHH*

AtRegR (NCBI AtRegR)
MSRKEQIIEVAMKLFAEKGYHATSMQEIAEHSRLAKGSLYNYFKSKEEIVLSIFQYHYDQLFQQFARIASDRSLTAREKFLKQLSLQIEAFEKH
KEIVQMHMGDHAQKVSEDVHALVLRIRSHLFDWYIQSFIDMYGERIRPFVLDCAIMLNGMLKEYLFFALFEKQSFPFQRLAPFLMERLDALVDS
LQHDMPLIRHDAKAEKARALALIDEMIEEARDDHTMDLLKQLKEEMERDEPRKAIVEAFLLYLQQSDMRQFVPALRAALALLEHHHHHH*

TmRegR (NCBI WP_004080830)
MSSETRRKILEAARKAFSKYGYDGVSMEEIAREAGVKKALIYYYFPSKDKLFEEVWREALEELESHLFAVTEETNSYFAKIKKFLKSYVDFVLN
KTVLNEIIEKEKSTVRFEEEKWSKLRERYESFIKRVEELIEEGKKQNYVYKDLDSRAAAELIVNSFGDVPKDKRLLQNIQEMILRGLLNVKTEE
GRLEHHHHHH*

VAO (NCBI P56216)
MSKTQEFRPLTLPPKLSLSDFNEFIQDIIRIVGSENVEVISSKDQIVDGSYMKPTHTHDP**H**HVMDQDYFLASAIVAPRNVADVQSIVGLANKFS
FPLWPISIGRNS**G**YGGAAPRVSGSVVLDMGKNMNRVLEVNVEGAYCVVEPGVTYHDLHNYLEANNLRDKLWLDVP**DL**GGGSVLGNAVERGVG**Y**T
PYGDHWMMHSGMEVVLANGELLRTGMGALPDPKRPETMGLKPEDQPWSKIAHLFPYGFGPYIDGLFSQSNMGIVTKIGIWLMPNPRGYQSYLIT
LPKDGDLKQAVDIIRPLRLGMALQNVPTI**R**HILLDAAVLGDKRSYSSRTEPLSDEELDKIAKQLNLGRWNFYGALYGPEPIRRVLWETIKDAFS
AIPGVKFYFPEDTPENSVLRV**R**DKTMQGIPTYDELK**W**IDWLPNGA**H**LFFSPIAKVSGEDAMMQYAVTKKRCQEAGLDFIGTFTVGMREMHHIVC
IVFNKKDLIQKRKVQWLMRTLIDDCAANGWGEYRTHLAFMDQIMETYNWNNSSFLRFNEVLKNAVDPNGIIAPGKSGVWPSQYSHVTWKLLEHH
HHHH*

TOYE (NCBI 3KRU)
MSILHMPLKIKDITIKNRIMMSPMCM**Y**SASTDGMPNDWHIVHYATRAIGGVGLIMQEATAVESRGR**I**TDHDLGIWNDEQVKELKKIVDICKANG
AVMGIQLAHAGRKCNISYEDVVGPSPIKAGDRYKLPRELSVEEIKSIVKAFGEAAKRANLAGYDVVEIHAAHG**Y**LIHEFLSPLSNKRKDEYGNS
IENRARFLIEVIDEVRKNWPENKPIFVRVSADDYMEGGINIDMMVEYINMIKDKVDLIDVSS**G**GLLNVDINLYPGYQVKYAETIKKRCNIKTSA
VGLITTQELAEEILSNERADLVALGRELLRNPYWVLHTYTSKEDWPKQYERAFKKLEHHHHHH*

EncP (NCBI AAF81735)

MTFVIELDMNVTLDQLEDAARQRTPVELSAPVRSRVRASRDVLVKFVQDERVI**Y**GVNTSMGGFVDHLVPVSQARQLQENLINAVATNVGAYLDD
TTARTIMLSRIVSLARGNSAITPANLDKLVAVLNAGIVPCIPEKGSLGTSGDLGPLAAIALVCAGQWKARYNGQIMPGRQALSEAGVEPMELSY
KDGLALI**N**GTSGMVGLGTMVLQAARRLVDRYLQVSALSVEGLAGMTKPFDPRVHGVKPHRGQRQVASRLWEGLADSHLAVNELDTEQTLAGEMG
TVAKAGSLAIEDAYSIKCTPQILGPVVDVLDRIGATLQDELNSSNDNPIVLPEEAEVFHNGHFHGQYVAMAMDHLNMALATVTNLANRRVDRFL
DKSNSNGLPAFLCREDPGLRLGLMGGQFMTASITAETRTLTIPMSVQSLTSTADFQDIVSFGFVAARRAREVLTNAAYVVAFELLCACQAVDIR
GADKLSSFTRPLYERTRKIVPFFDRDETITDYVEKLAADLIAGEPVDAAVAAHLEHHHHHH*

This journal is © The Royal Society of Chemistry 2024
Please do not adjust margins

Please do not adjust margins

Schemes

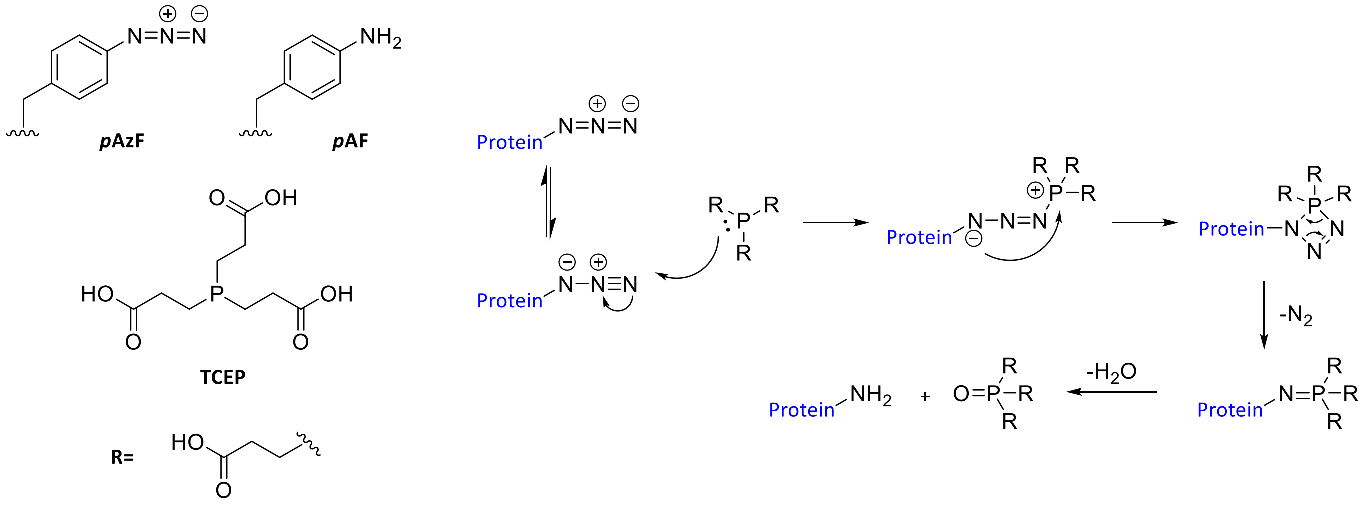

Scheme S1. Staudinger reduction of *p*AzF to *p*AF using Tris(2-carboxyethyl)phosphine (TCEP).

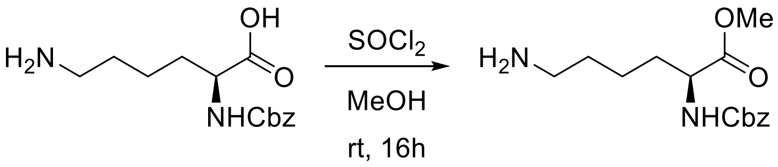

Scheme S2. Synthetic route for the synthesis of Cbz-*L*-Lys-OMe.

methyl (2s)-6-amino-2-(((benzyloxy)carbonyl)amino)hexanoate (Cbz-*L*-Lys-OMe)

Cbz-Lys-OH (25.00 g, 89.18 mmol, 1.00 eq) was dissolved in MeOH (270 mL, 0.33 M) and cooled to 0 °C. $SOCl_2$ (7.2 mL, 98.10 mmol, 1.10 eq) was added dropwise. The ice bath was removed, and the mixture was stirred at room temperature for 16 h. The reaction was monitored by TLC. After completion, the reaction mixture was concentrated to afford the crude product as a clear oil. The product was directly used in the next step without further purification.

Please do not adjust margins

Supporting table

Table S1. List of primers used in this study. Mutations are highlighted in red.

Entry	Name	Oligo DNA sequence (5‘)
1	LmrR_**V15TAG**_fw	GCT CAA ACC AAT TAG ATC CTG CTG AAT
2	LmrR_**V15TAG**_rv	ATT CAG CAG GAT CTA ATT GGT TTG AGC
3	LmrR_**M8TAG**_fw	C CCG AAA GAA TAG CTG CGT GCT CAA AC
4	LmrR_**M8TAG**_rv	CAG CTA TTC TTT CGG GAT TTC GGC ACC
5	LmrR_**M89TAG**_fw	AC TAG CGC CTG GCG TTC GAA TCC
6	LmrR_**M89TAG**_rv	GC CAG GCG CTA GTT TTC ATG GCC GAT TTC
7	LmrR_**V15K**_fw	GT GCT CAA ACC AAT AAA ATC CTG CTG AAT GTC
8	LmrR_**V15K**_rv	ATT GGT TTG AGC ACG CAG CAT TTC TTT CG
9	LmrR_**M8K**_fw	C CCG AAA GAA AAA CTG CGT GCT CAA ACC
10	LmrR_**M8K**_rv	G CAG TTT TTC TTT CGG GAT TTC GGC ACC
11	LmrR_**M89K**_fw	C AAA CGC CTG GCG TTC GAA TCC TG
12	LmrR_**M89K**_rv	CGC CAG GCG TTT GTT TTC ATG GCC GAT TTC
13	BbmrR_**S14TAG**_fw	GTT CGC GGG TAG ATA GAC ACA ATT ATT CTG
14	BbmrR_**S14TAG**_rv	T CTA CCC GCG AAC TAA GTC GCT GTT ATT G
15	BbmrR_**S14K**_fw	GTT CGC GGG AAA ATA GAC ACA ATT ATT CTG TC
16	BbmrR_**S14K**_rv	T TTT CCC GCG AAC TAA GTC GCT GTT ATT GAG
17	VAO_**H61T**_fw	CAC GAT CCA ACC CAC GTA ATG GAC CAA G
18	VAO_**H61T**_rv	G GGT TGG ATC GTG GGT GTG AGT AGG
19	VAO_**D170TAG**_fw	CCC TAG TTG GGC GGC GGG TCT G
20	VAO_**D170TAG**_rv	C GCC CAA CTA GGG TAC GTC TAA CCA AAG
21	VAO_**L171TAG**_fw	C GAT TAG GGC GGC GGG TCT GTG
22	VAO_**L171TAG**_rv	GCC GCC CTA ATC GGG TAC GTC TAAC
23	VAO_**Y187TAG**_fw	G GTC GGT TAG ACC CCT TAC GGT GAC
24	VAO_**Y187TAG**_rv	G GGT CTA ACC GAC CCC ACG CTC
25	VAO_**H422TAG**_fw	C AAC GGA GCC TAG CTG TTC TTC TCT CC
26	VAO_**H422TAG**_rv	G CTA GGC TCC GTT GGG TAA CCA ATC G
27	VAO_**D170K**_fw	CCCAAATTGGGCGGCGGGTCTG
28	VAO_**D170K**_rv	CGCCCAATTTGGGTACGTCTAACCAAAG
29	VAO_**L171K**_fw	CC GAT AAA GGC GGC GGG TCT GTG
30	VAO_**L171K**_rv	CC GCC TTT ATC GGG TAC GTC TAA CC
31	VAO_**Y187K**_fw	G GTC GGT AAA ACC CCT TAC GGT GAC
32	VAO_**Y187K**_rv	GG GGT TTT ACC GAC CCC ACG CTC
33	VAO_**H422K**_fw	C AAC GGA GCC AAA CTG TTC TTC TCT CC
34	VAO_**H422K**_rv	G TTT GGC TCC GTT GGG TAA CCA ATC G
35	TOYE_**Y27TAG**_fw	G TAG AGC GCC AGT ACC GAT GGG ATG
36	TOYE_**Y27TAG**_rv	ACT GGC GCT CTA CAT ACA CAT GGG TGA C
37	TOYE_**I67TAG**_fw	CGC GGT CGT TAG ACA GAT CAT GAT CTT GG

 This journal is © The Royal Society of Chemistry 2024

Please do not adjust margins

38	TOYE_**I67TAG**_rv	T CTA ACG ACC GCG AGA TTC TAC TGC CGT TG
39	TOYE_**Y168TAG**_fw	CG GCT CAC GGC TAG CTG ATA CAT GAA TTT C
40	TOYE_**Y168TAG**_rv	G CTA GCC GTG AGC CGC ATG GAT TTC
41	TOYE_**Y27K**_fw	G AAA AGC GCC AGT ACC GAT GGG ATG
42	TOYE_**Y27K**_rv	ACT GGC GCT TTT CAT ACA CAT GGG TGAC
43	TOYE_**I67K**_fw	CGC GGT CGT AAA ACA GAT CAT GAT CTT GG
44	TOYE_**I67K**_rv	T TTT ACG ACC GCG AGA TTC TAC TGC CGT TG
45	TOYE_**Y168K**_fw	CG GCT CAC GGC AAA CTG ATA CAT GAA TTT C
46	TOYE_**Y168K**_rv	G TTT GCC GTG AGC CGC ATG GAT TTC
47	EncP_**Y54TAG**_fw	GAA CGG GTA ATA TAG GGC GTA AAC ACA TCC
48	EncP_**Y54TAG**_rv	ATA TTA CCC GTT CAT CCT GTA CGA ATT TAA CCA AC
49	EncP_**N196TAG**_fw	G CAA GCG TAG CGT AGA TTG GTC GAT CG
50	EncP_**N196TAG**_rv	ACG CTA CGC TTG CAG CAC CAT TGT TCC
51	EncP_**Y54K**_fw	G CCG AAA TCA CAA GCG CGG CAG C
52	EncP_**Y54K**_rv	GC TTG TGA TTT CGG CAC CAG ATG ATC AAC
53	EncP_**N196K**_fw	G CAA GCG AAA CGT AGA TTG GTC GAT CG
54	EncP_**N196K**_rv	ACG TTT CGC TTG CAG CAC CAT TGT TCC

Please do not adjust margins

Supporting Figures

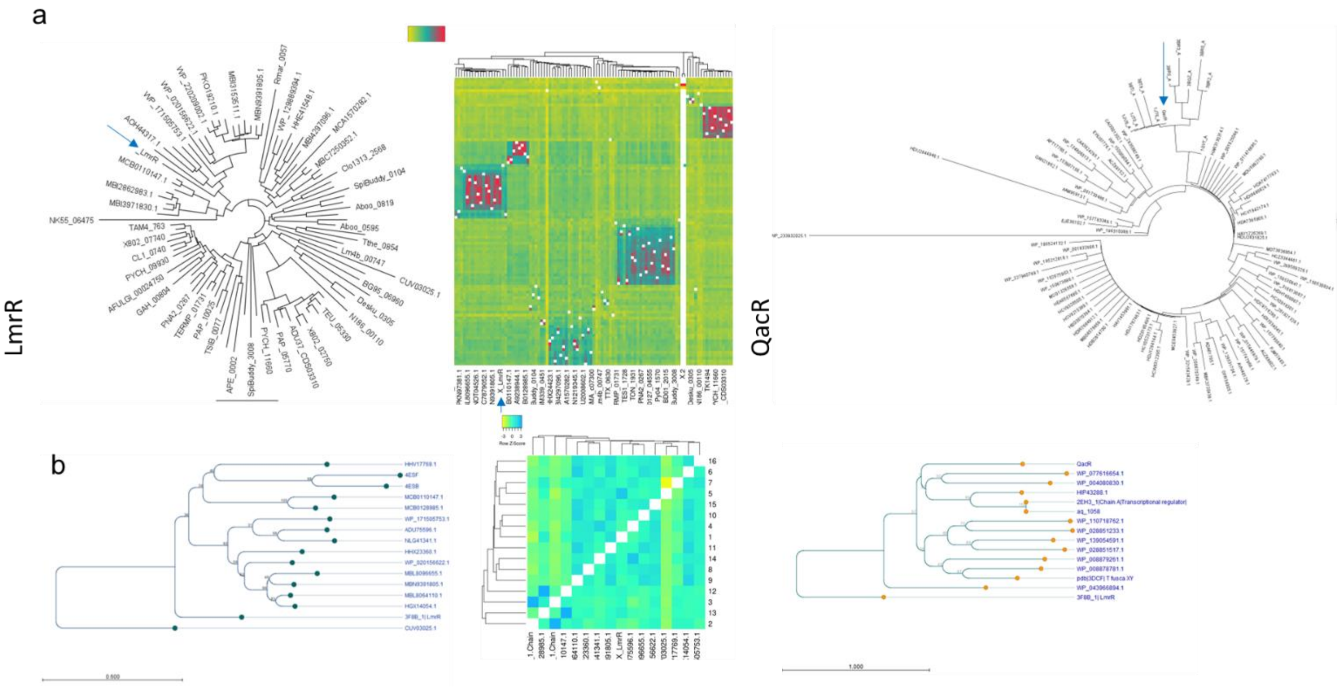

Figure S1. Pocket-guided search of scaffolds. LmrR and QacR were used as sequence templates. After an initial search, aberrant sequences were manually discarded. Redundant sequences were discarded with a 90% cut-off. Sequence diversity was analyzed by a cladogram. Templates are indicated with a blue arrow.

This journal is © The Royal Society of Chemistry 2024

Please do not adjust margins

SDS-PAGE analysis

For SDS-PAGE analysis of all samples a PageRuler™ Prestained Protein Ladder (Thermo Scientific™) marker from 10 to 180 kDa was used. For all SDS-PAGEs, abbreviations stands for: M, Marker; CE, cell-extract; CFE, cell-free extract; FT, flow-through; W, wash with 40 mM imidazole, E, elution fraction and C, concentrated sample, IP, input.

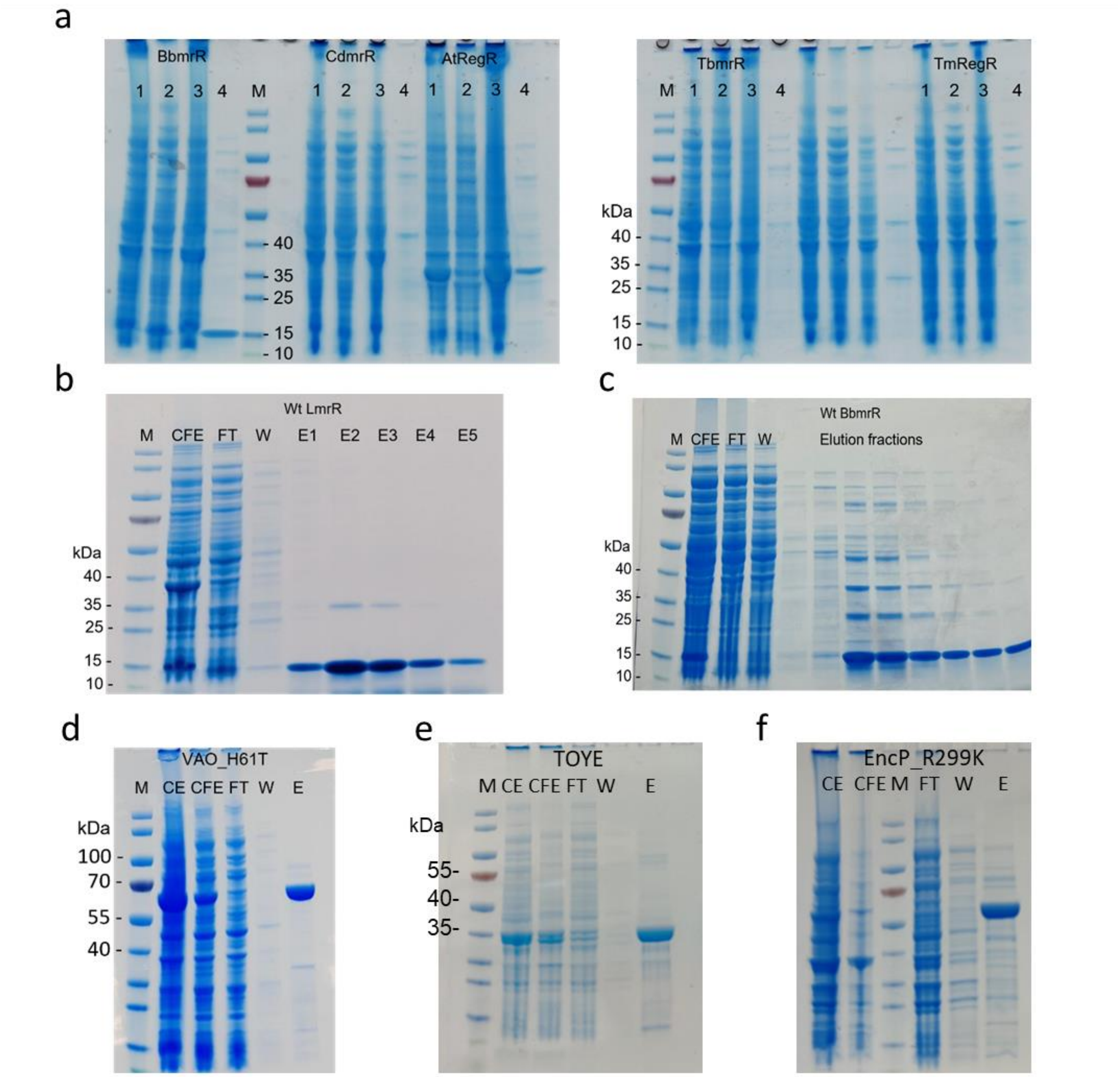

Figure S2. SDS-PAGE of selected scaffolds. Purifications of scaffolds were done by immobilized metal affinity chromatography (Ni^{+2}-NTA) and affinity purification for LmrR (Strep-tag II). a) Proteins identified from pocket-guided search, BbmrR (MW 15 kDa), CdmrR (MW 15 kDa), AtRegR (MW 30 kDa), TmbrR (MW 15 kDa) and TmRegR (MW 30 kDa), 1: CE, 2: CFE, 3: insoluble fraction and 4: E. b) LmrR, MW 15 kDa, c) BbmrR MW 15 kDa, d) VAO_H61T, MW 64 kDa, e) TOYE, MW 38 kDa and f) EncP_R299K, MW 57 kDa.

Please do not adjust margins

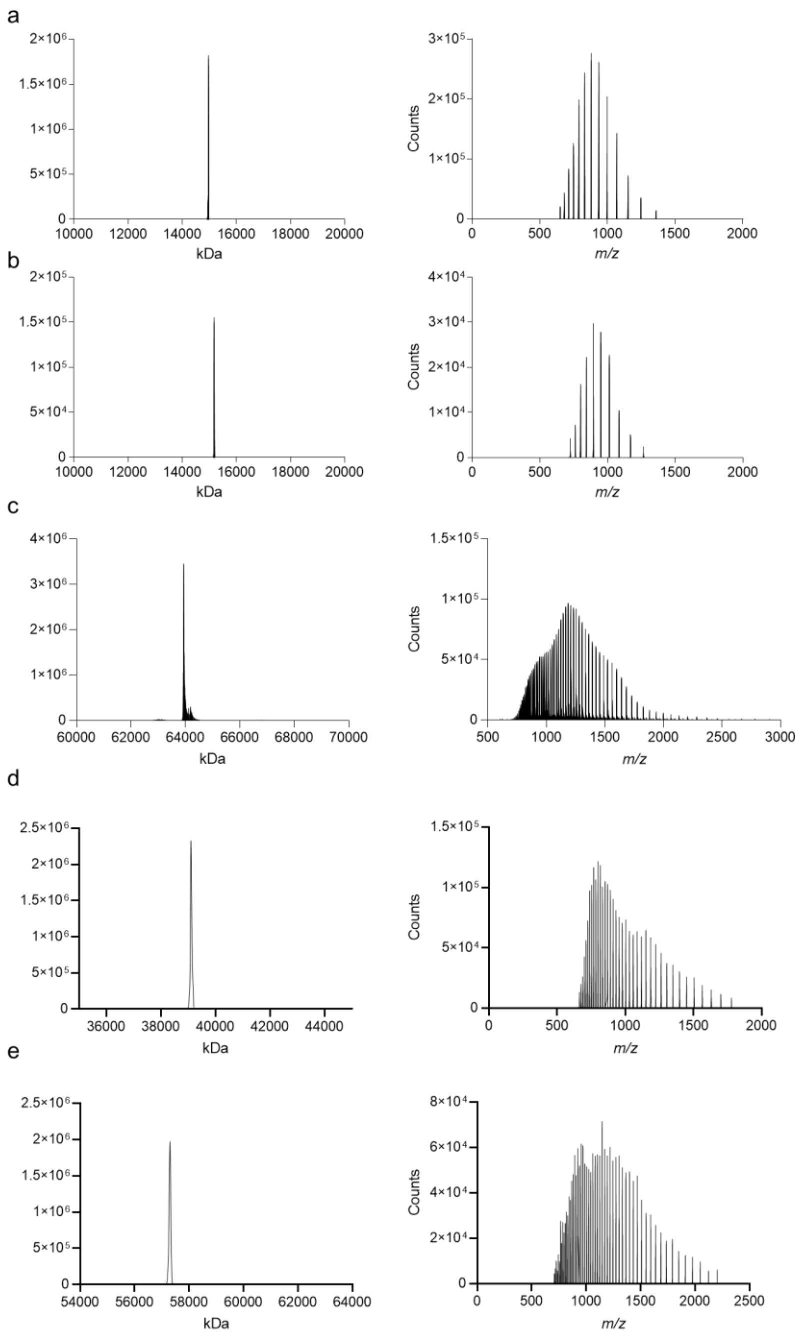

Figure S3. LC-MS analysis of selected scaffolds. Mass spectrum of components (left panel) and deconvoluted ion set (right panel) of each purified scaffold. a) LmrR, M_{calc}: 15,102 Da; M_{obs}: 14,973 Da. Difference in mass corresponds to N-terminal methionine cleavage (Δ129 Da). b) BbmrR, M_{calc}: 15,181 Da; M_{obs}: 15,182 Da. c) VAO_H61T, M_{calc}: 64,064 Da; M_{obs}: 63,934 Da. Difference in mass corresponds to N-terminal methionine cleavage (Δ129 Da). d) TOYE, M_{calc}: 39,238 Da; M_{obs}: 39,110. Difference in mass corresponds to N-terminal methionine cleavage (Δ128 Da) and e) EncP_R299K. $M_{calc:}$ 57,453; $M_{obs:}$ 57,320. Difference in mass corresponds to N-terminal methionine cleavage (Δ133 Da).

 This journal is © The Royal Society of Chemistry 2024

Please do not adjust margins

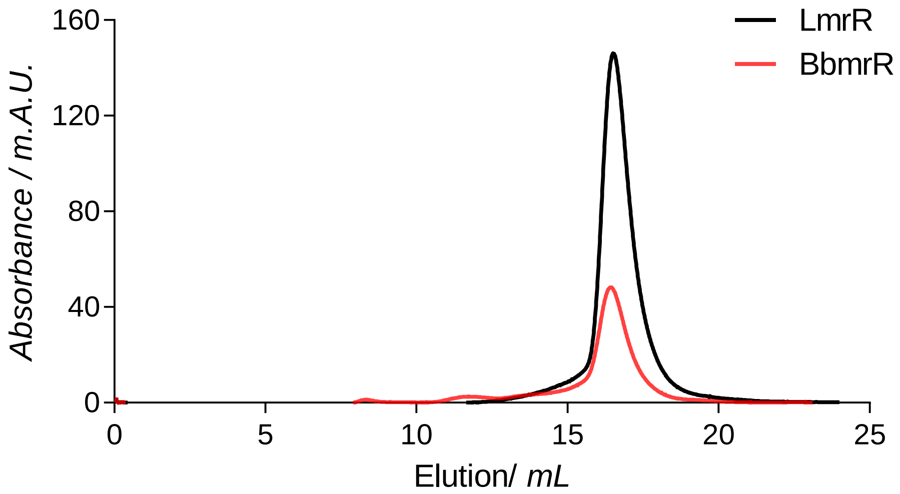

Figure S4. Size-exclusion chromatography of BbmrR. The oligomeric state of BbmrR (red line) was determined via analytical size-exclusion chromatography on a Superdex 200 10/300 gel filtration column. For comparison, LmrR (black line), described as dimer ca. 30 kDa,[21] was evaluated in the same conditions.

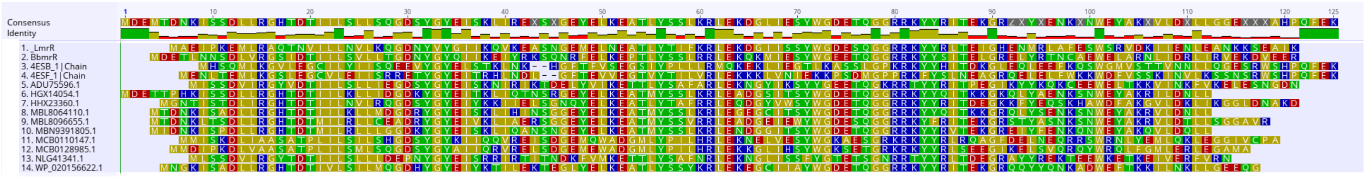

Figure S5. Multiple sequence alignment. Preliminary amino acidic sequence analysis was performed using the sequence of LmrR and a selected set of LmrR-like proteins. Analysis was performed MUltiple Sequence Comparison by Log-Expectation (MUSCLE) algorithm using Geneious Prime® 2021.2.2. with ClustalW and UPGMB iteration methods.

Please do not adjust margins

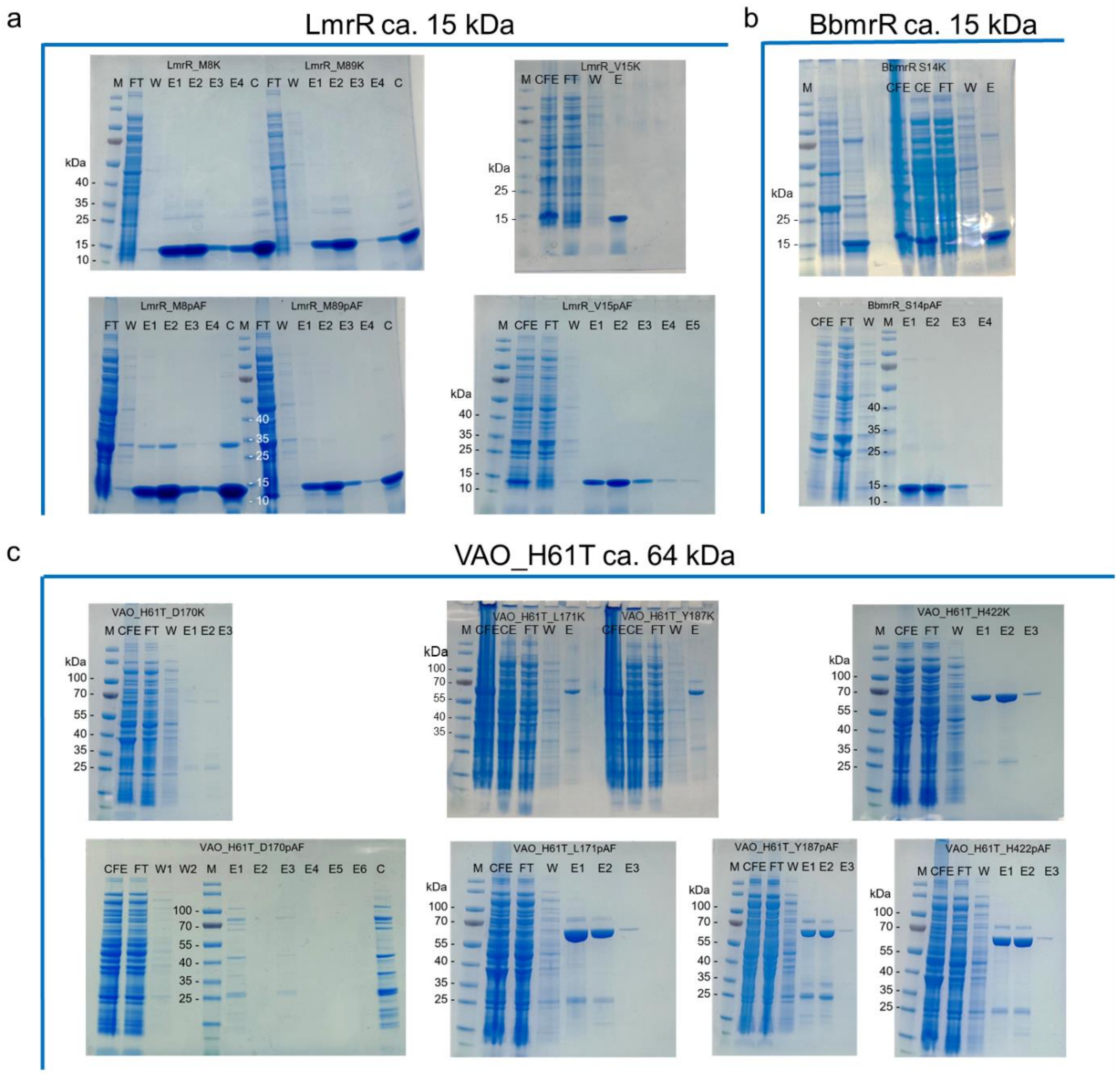

Figure S6. SDS-PAGE of lysine and *p*AzF variants. Purifications of scaffolds were done by immobilized metal affinity chromatography (Ni^{+2}-NTA) and affinity purification for LmrR variants (Strep-tag II) a) LmrR variants ca. MW 15 kDa, b) BbmrR MW 15 kDa, c) VAO_H61T, MW 64 kDa, d) EncP_R299K, MW 57 kDa and TOYE, MW 38 kDa.

This journal is © The Royal Society of Chemistry 2024

Please do not adjust margins

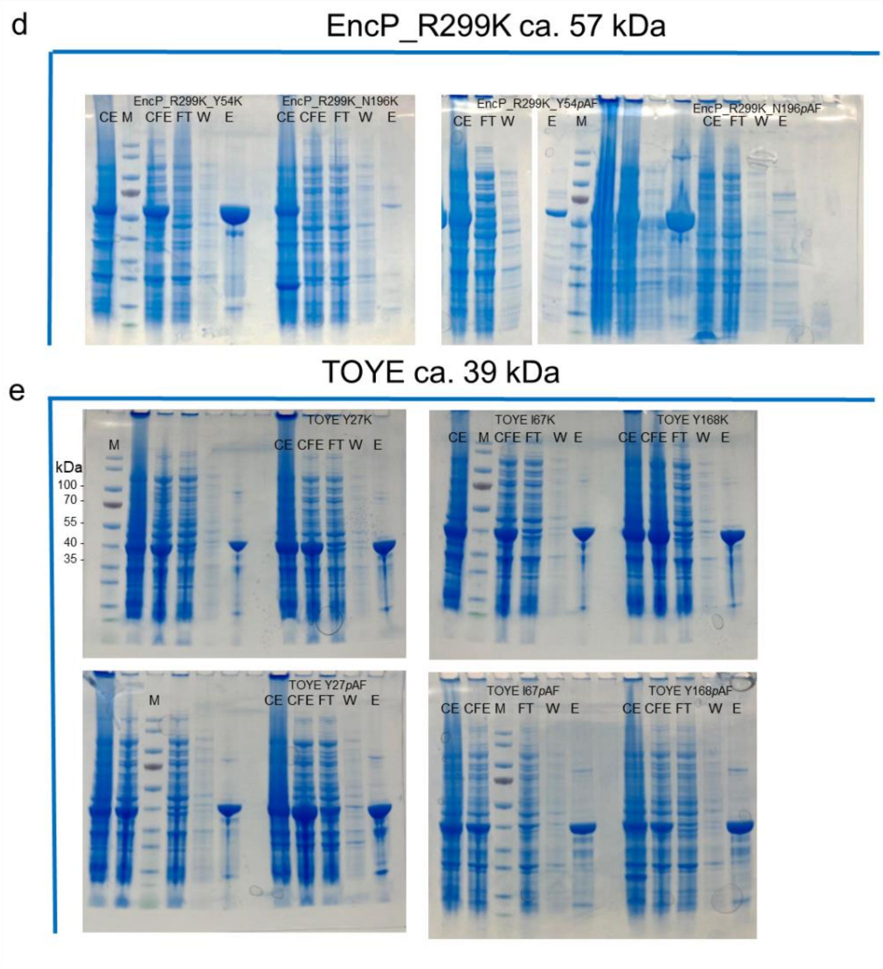

Figure S6. SDS-PAGE of lysine and *p*AzF variants. Continuation.

Please do not adjust margins

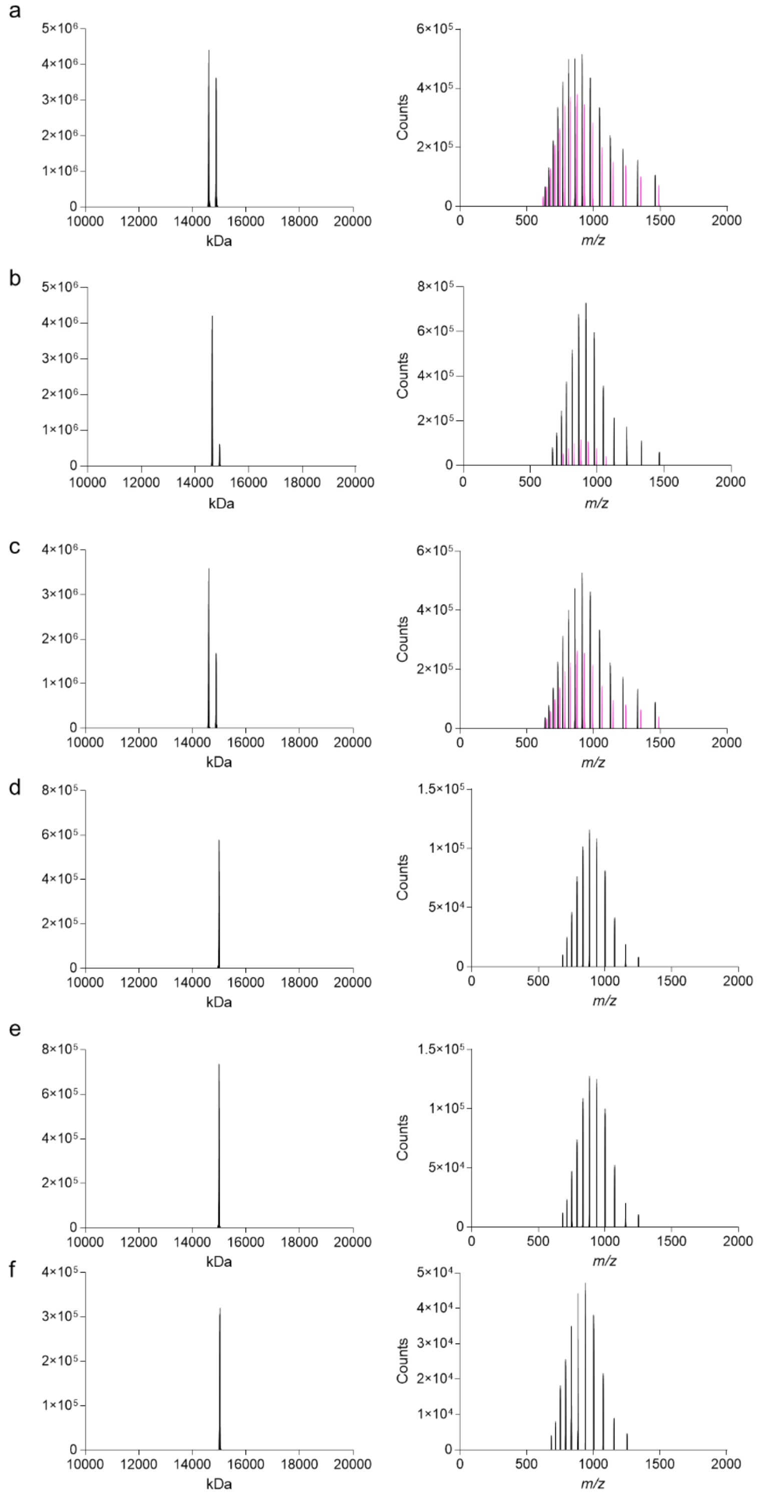

Figure S7. LC-MS analysis of lysine and *p*AF LmrR variants. Mass spectrum of components (left panel) and deconvoluted ion set (right panel) of each purified scaffold. a) LmrR_M8K. M_{calc}: 15,025 Da; M_{obs}: 14,617 Da. Difference in mass between peaks corresponds to N-terminal methionine and histidine cleavage (Δ 407Da). b) LmrR_V15K. M_{calc}: 15,057 Da; M_{obs}: 14,652 Da. Difference in mass between peaks corresponds to N-terminal methionine and histidine cleavage (Δ407Da). c) LmrR_M89K. M_{calc}: 15,025 Da; M_{obs}: 14,617 Da. Difference in mass between peaks corresponds to N-terminal methionine and histidine cleavage (Δ407Da). d) LmrR_M8*p*AF. M_{calc}: 15,133 Da; M_{obs}: 15,002 Da. Difference in mass corresponds to N-terminal methionine cleavage (Δ129 Da). e) LmrR_V15*p*AF. M_{calc}: 15,165 Da; M_{obs}: 15,033 Da. Difference in mass corresponds to N-terminal methionine cleavage (Δ132 Da). e) LmrR_M89*p*AF. M_{calc}: 15,133 Da; M_{obs}: 15,000 Da. Difference in mass corresponds to N-terminal methionine cleavage (Δ132 Da).

This journal is © The Royal Society of Chemistry 2024

Please do not adjust margins

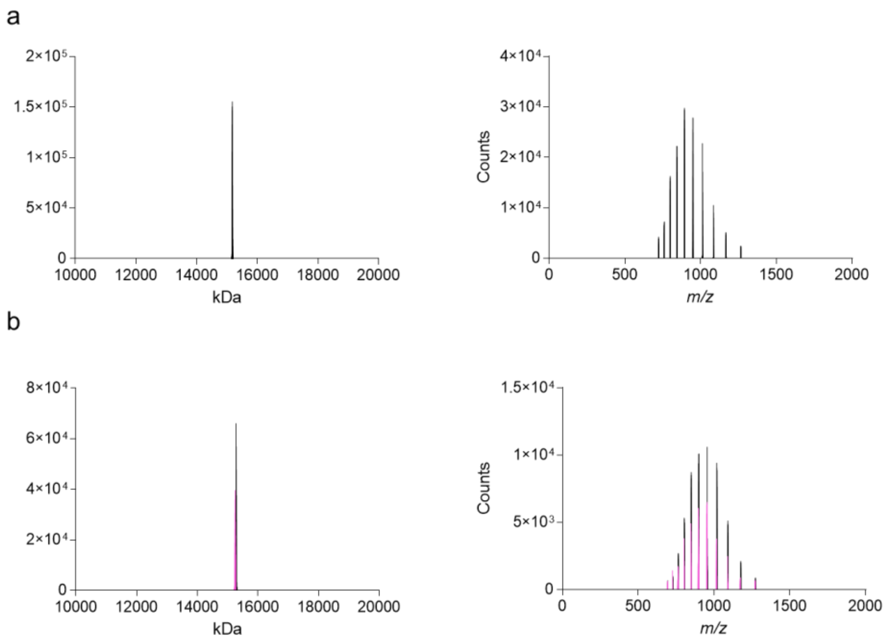

Figure S8. LC-MS analysis of lysine and *p*AF BbmrR variants. Mass spectrum of components (left panel) and deconvoluted ion set (right panel) of each purified scaffold. BbmrR_S14K. M_{calc}: 15,222 Da; M_{obs}: 15,219 Da. BbmrR_S14*p*AF. M_{calc}: 15,256 Da ; M_{obs}: 15,283 Da. The difference in mass between the two peaks corresponds to an incomplete reduction of *p*AzF to *p*AF (Δ26 Da).

Please do not adjust margins

Please do not adjust margins

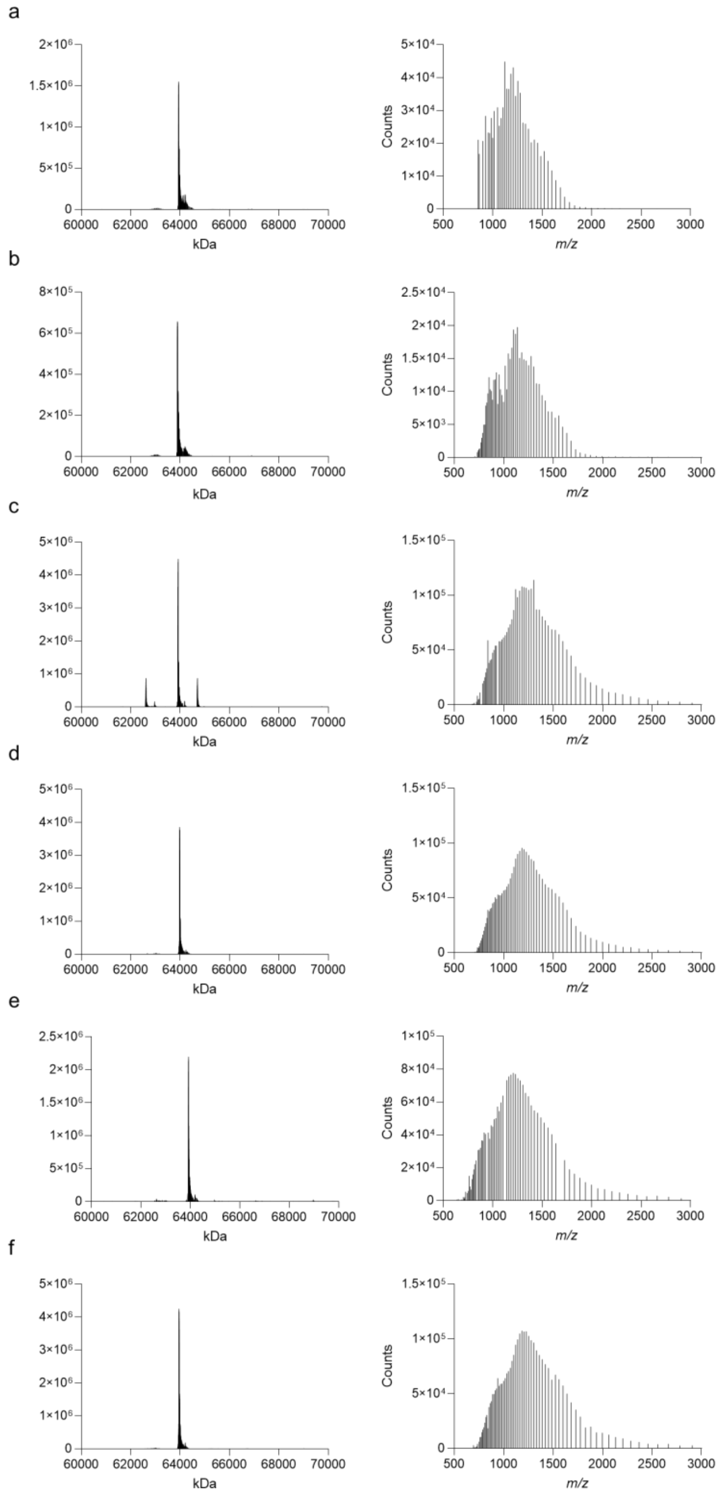

Figure S9. LC-MS analysis of lysine and *p*AF VAO_H61T variants. Mass spectrum of components (left panel) and deconvoluted ion set (right panel) of each purified scaffold. a) VAO_H61T_L171K. M_{calc}: 64,079 Da; M_{obs}: 63,949 Da. Difference in mass corresponds to N-terminal methionine cleavage (Δ130 Da). b) VAO_H61T_Y187K. M_{calc}: 64,029 Da; M_{obs}: 63,899 Da. Difference in mass corresponds to N-terminal methionine cleavage (Δ130 Da). c) VAO_H61T_H422K. M_{calc}: 64,055 Da; M_{obs}: 63,925 Da. Difference in mass corresponds to N-terminal methionine cleavage (Δ130 Da). d) VAO_H61T_L171*p*AF. M_{calc}: 64,113 Da; M_{obs}: 63,983 Da. Difference in mass corresponds to N-terminal methionine cleavage (Δ130 Da). e) VAO_H61T_Y187*p*AF. M_{calc}: 63932 Da; M_{obs}: 63,933 Da. VAO_H61T_H422*p*AF. M_{calc}: 64,089 Da; M_{obs}: 63,959 Da. Difference in mass corresponds to N-terminal methionine cleavage (Δ130 Da).

This journal is © The Royal Society of Chemistry 2024

Please do not adjust margins

Please do not adjust margins

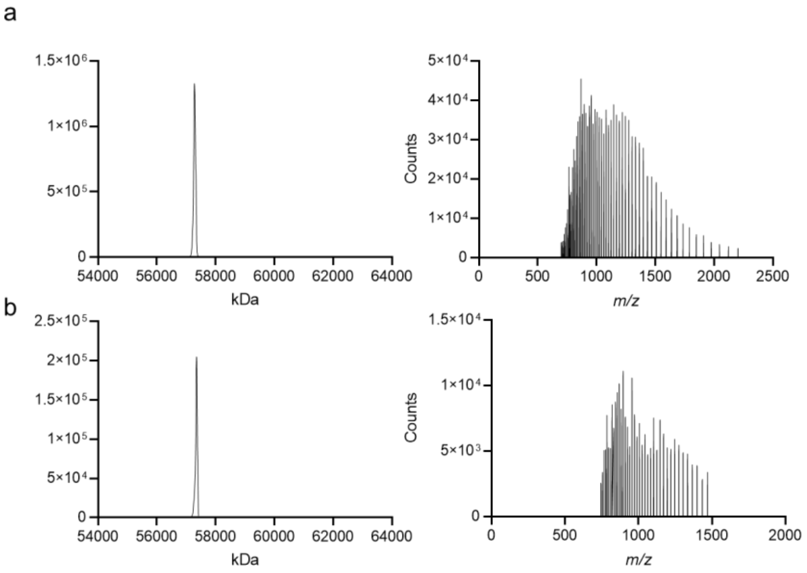

Figure S10. LC-MS analysis of lysine and *p*AF EncP_R299K variants. Mass spectrum of components (left panel) and deconvoluted ion set (right panel) of each purified scaffold. a) EncP_R299K_Y54K. M_{calc}: 57,418 Da; M_{obs}: 57,290 Da. Difference in mass corresponds to N-terminal methionine cleavage (Δ128 Da). b) EncP_R299K_N196K M_{calc}: 57,467 Da; M_{obs}:57,339 Da. Difference in mass corresponds to N-terminal methionine cleavage (Δ128 Da).

Please do not adjust margins

Please do not adjust margins

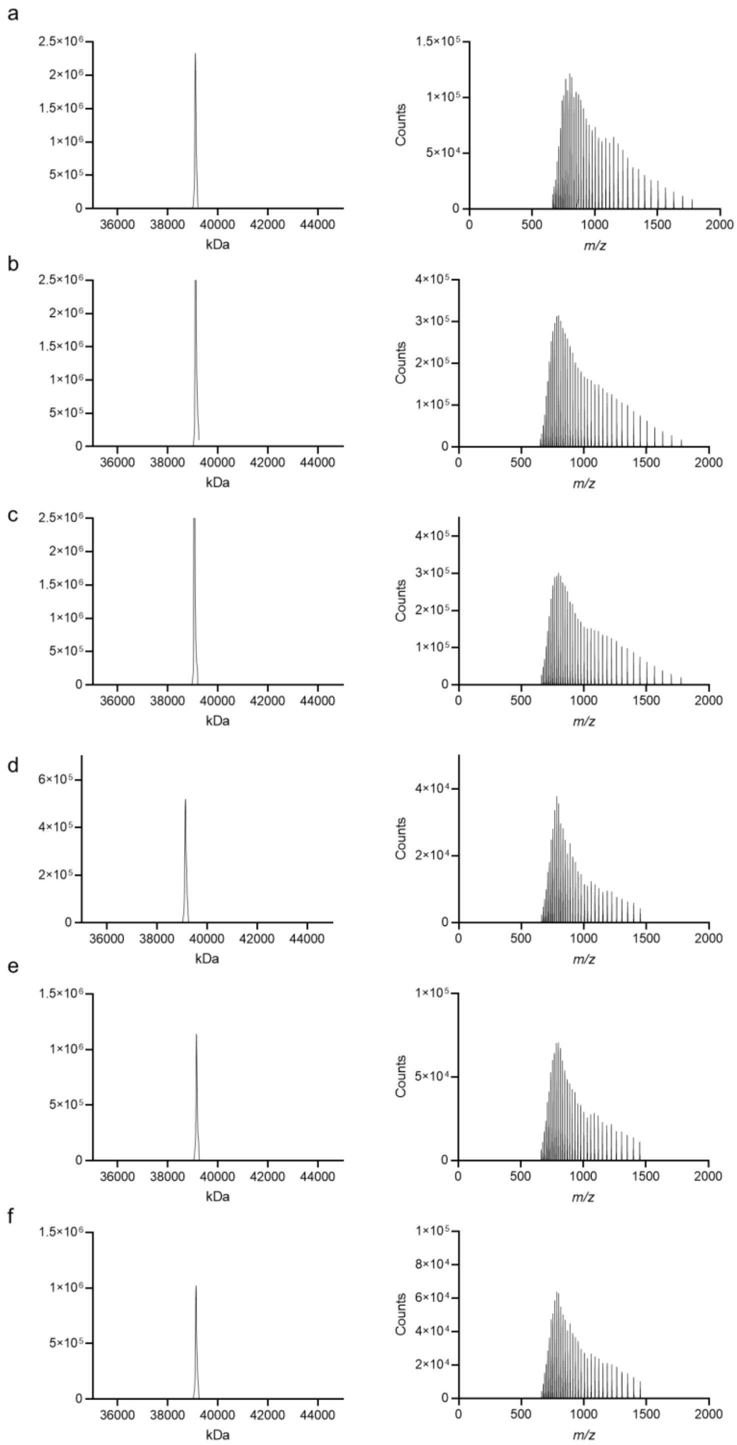

Figure S11. LC-MS analysis of lysine and *p*AF TOYE variants. Mass spectrum of components (left panel) and deconvoluted ion set (right panel) of each purified scaffold. a) TOYE_Y27K M_{calc}: 39,203 Da; M_{obs}: 39,075 Da. Difference in mass corresponds to N-terminal methionine cleavage (Δ128 Da). b) TOYE_I67K M_{calc}: 39,253 Da; M_{obs}: 39,122 Difference in mass corresponds to N-terminal methionine cleavage (Δ131 Da). c) TOYE_Y168K M_{calc}: 39,203 Da; M_{obs}: 39,077 Difference in mass corresponds to N-terminal methionine cleavage (Δ130 Da). d) TOYE_Y27*p*AF M_{calc}: 39,237 Da; M_{obs}: 39,109 Da. Difference in mass corresponds to N-terminal methionine cleavage (Δ128 Da). e) TOYE_I67*p*AF M_{calc}: 39,287 Da; M_{obs}: 39,154 Difference in mass corresponds to N-terminal methionine cleavage (Δ131 Da). f) TOYE_Y168*p*AF M_{calc}: 39,207 Da; M_{obs}: 39,072 Difference in mass corresponds to N-terminal methionine cleavage (Δ131 Da).

This journal is © The Royal Society of Chemistry 2024

Please do not adjust margins

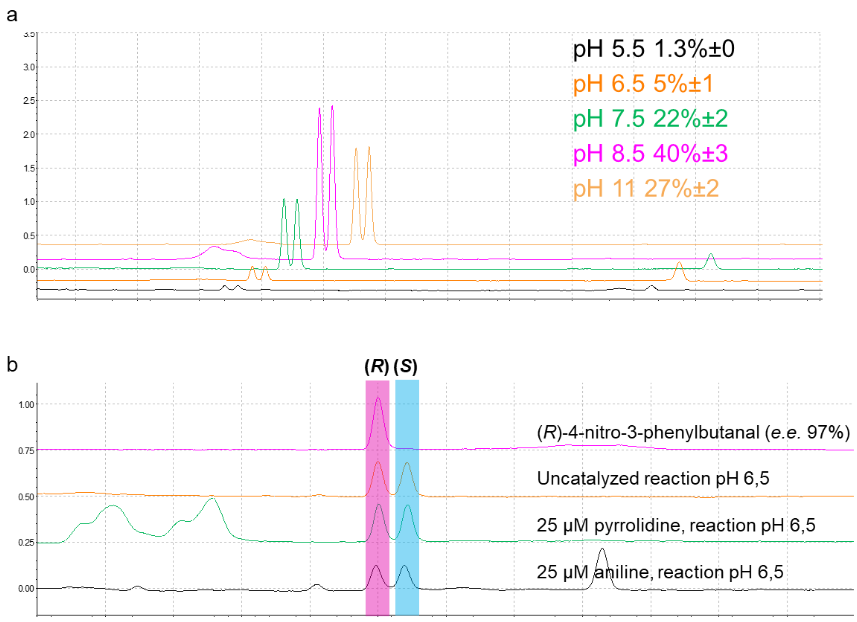

Figure S12. **Preliminary small-scale conversion optimization.** a) Reactions were set at pH 5.5-11 in 50 mM HEPES 150 mM NaCl solution. b) Qualitative representation of reaction using 25 µM of pyrrolidine or aniline as side-chain catalysts controls. Reactions were formulated with 1 mM *E*-cinnamaldehyde and 50 mM nitromethane.

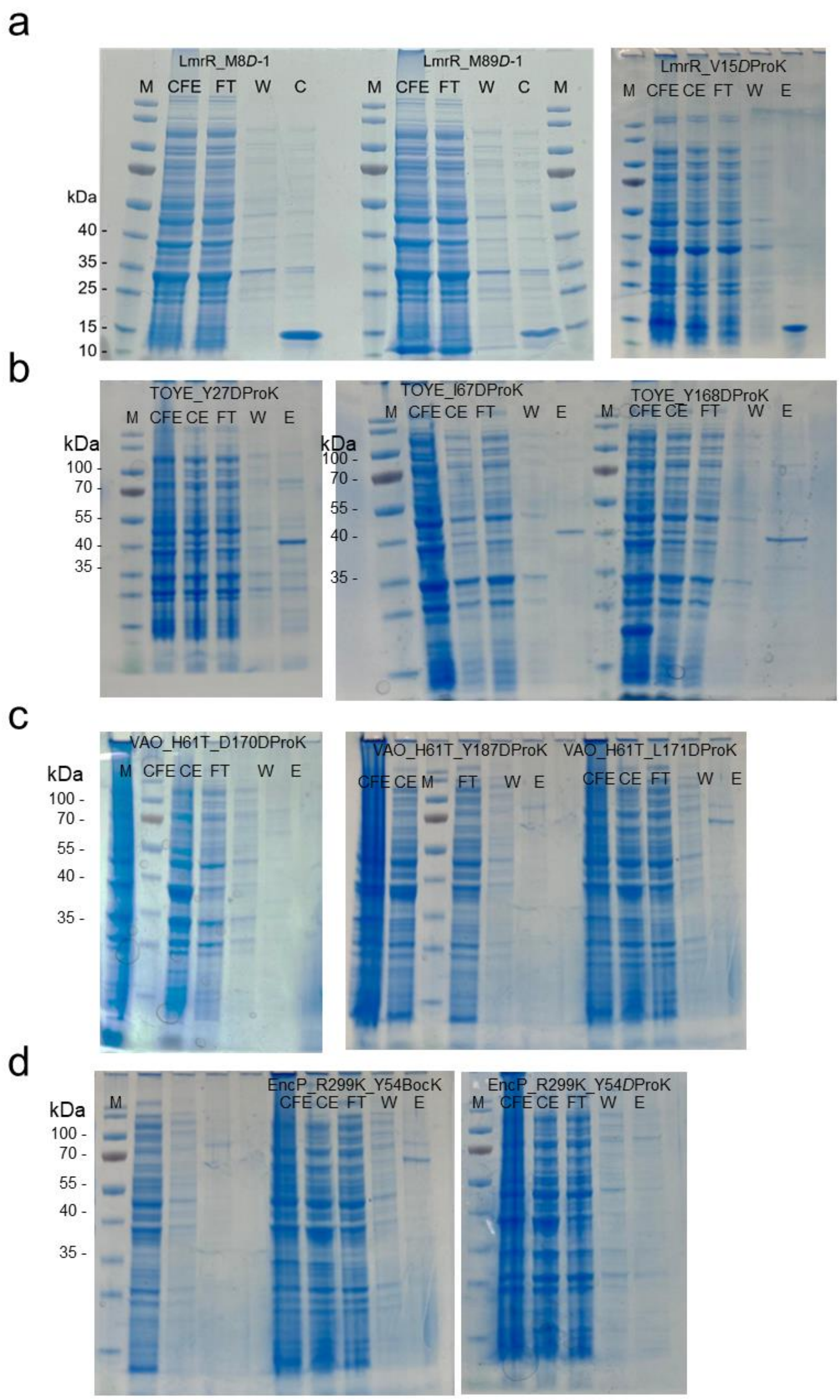

Figure S13. SDS-PAGE of *D*ProK variants. Purifications of scaffolds were done by immobilized metal affinity chromatography (Ni^{+2}-NTA) and affinity purification for LmrR variants (Strep-tag II): a) LmrR variants ca. MW 15 kDa, b) TOYE variants MW 38 kDa, c) VAO_H61T variants, MW 64 kDa and d) EncP_R299K variants.

This journal is © The Royal Society of Chemistry 2024

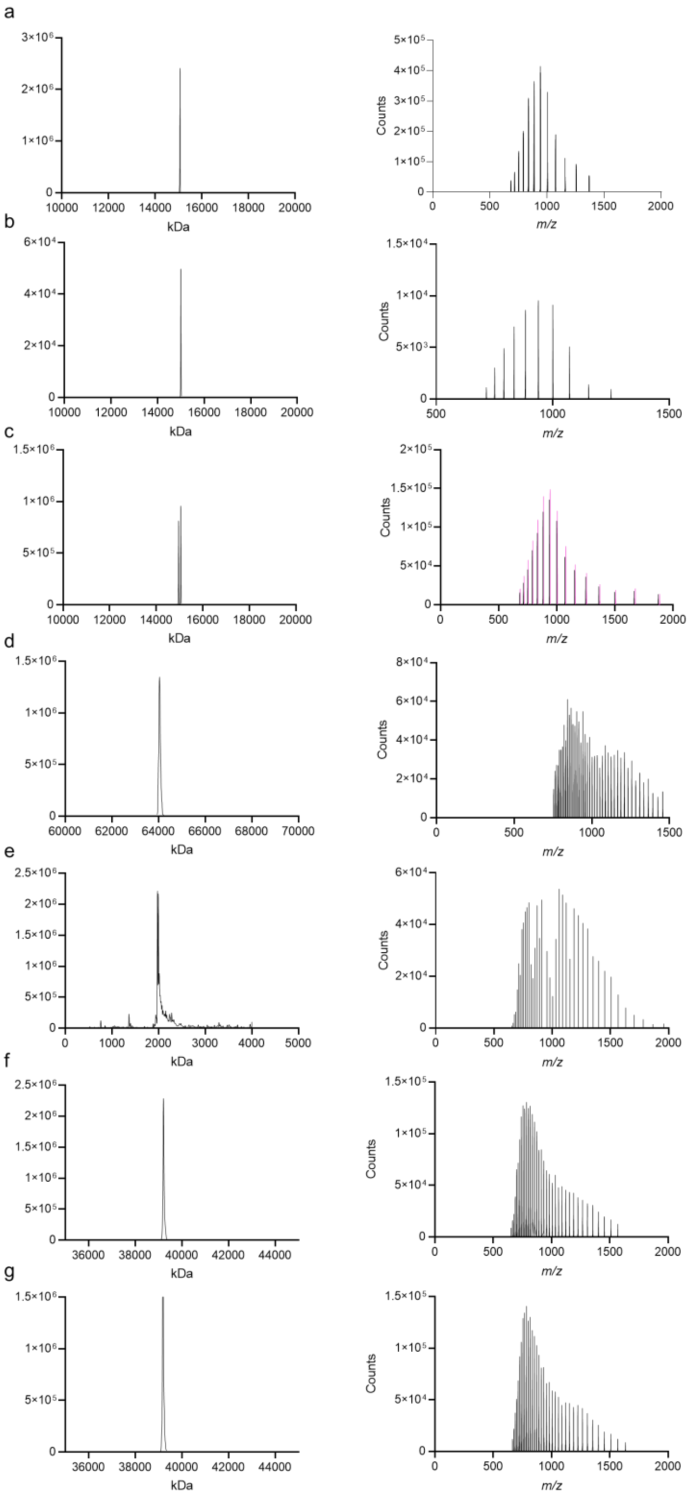

Figure S14. LC-MS analysis of *D*ProK variants. Mass spectrum of components (left panel) and deconvoluted ion set (right panel) of each purified scaffold. a) LmrR_M8*D*ProK, M_{calc}: 15,196 Da; M_{obs}: 15,060 Da. Difference in mass corresponds to N-terminal methionine cleavage (Δ134 Da). b) a) LmrR_V15*D*ProK, M_{calc}: 15,155 Da; M_{obs}: 14,748Da. Difference in mass corresponds to N-terminal methionine and 2x histidine cleavage (Δ407Da). c) LmrR_M89*D*ProK M_{calc}: 15.196 Da; M_{obs}: 15,060Da. Difference in mass corresponds to N-terminal methionine cleavage (Δ129 Da). d) VAO_H61T_L171*D*ProK, M_{calc}: 64,212 Da; M_{obs}: 64,048 Da, difference in mass corresponds to N-terminal methionine cleavage (Δ129 Da). e) TOYE_Y27*D*ProK, M_{calc}: 39,300 Da; M_{obs}: 39,168, difference in mass corresponds to N-terminal methionine cleavage (Δ132 Da) f) TOYE_I67*D*ProK, M_{calc}: 39,350 Da; M_{obs}: 39,214 difference in mass corresponds to N-terminal methionine cleavage (Δ136 Da) g) TOYE_Y168*D*ProK, M_{calc}: 39,300 Da; M_{obs}: 34,172 difference in mass corresponds to N-terminal methionine cleavage (Δ128 Da).

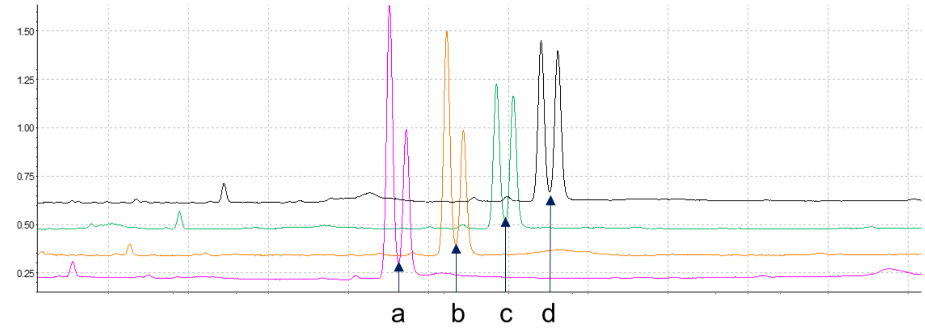

Figure S15. TOYE as biocatalyst for Michael addition reaction. Chromatograms of small-scale conversions were performed using 25 µM TOYE, a) and b) I67*D*ProK or c) and d) Y168 *D*ProK at pH 6.5 in 50 mM HEPES and 150 mM NaCl buffer. Reactions were set with 1 mM *E*-cinnamaldehyde and a) and c) 50 or b) and d) 100 mM nitromethane as nucleophile. Chromatograms were manually shifted to facilitate qualitative comparison.

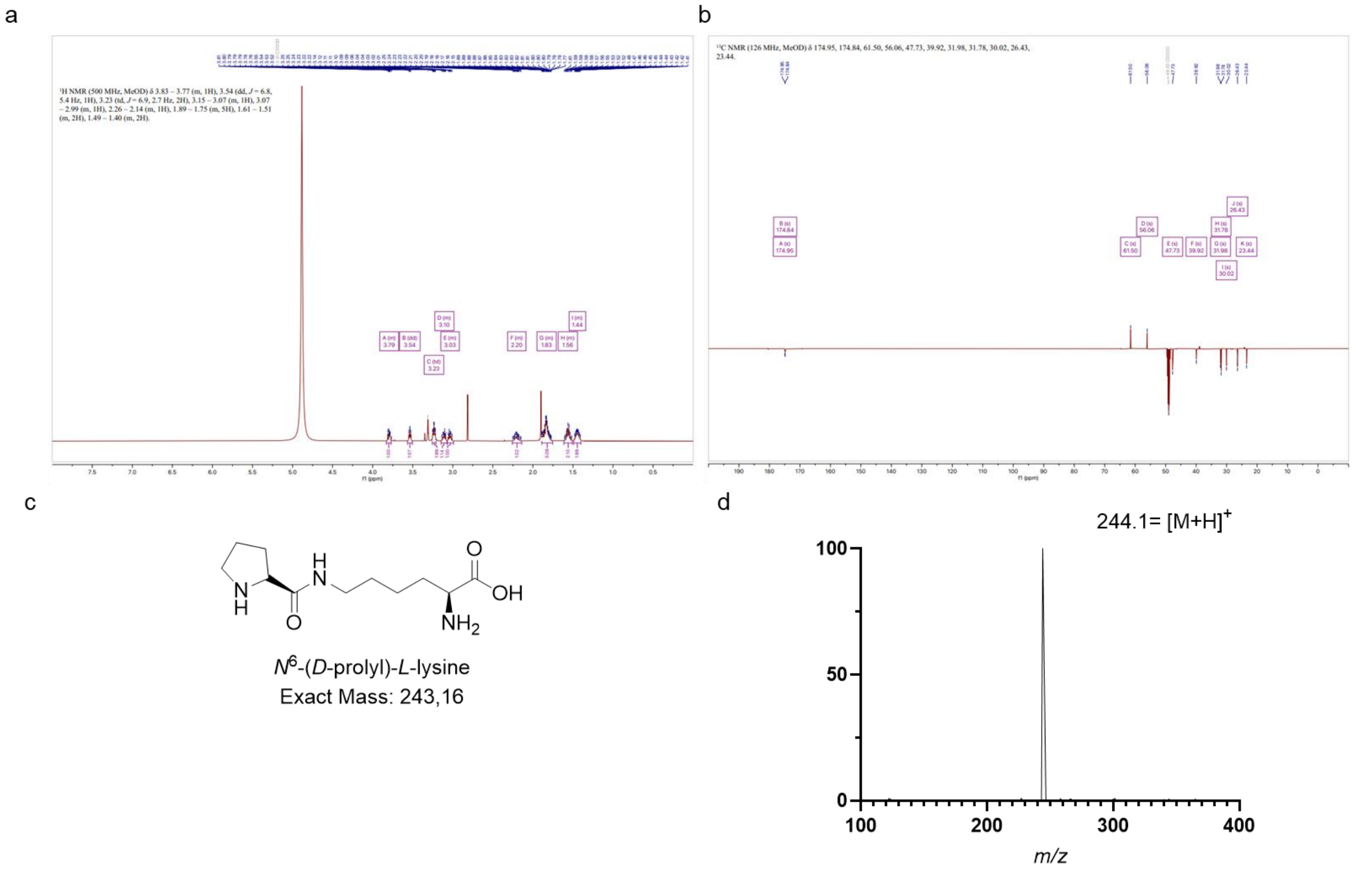

Figure S16. Analysis of synthesized N^6-prolyl-*L*-lysine. Synthesis of ncAA *D*ProK was analysed by a) hydrogen and b) carbon NMR, d) LC-MS, and specific rotation (1 g 100 mL^{-1} displayed +40 and +38).

 This journal is © The Royal Society of Chemistry 2024

Please do not adjust margins

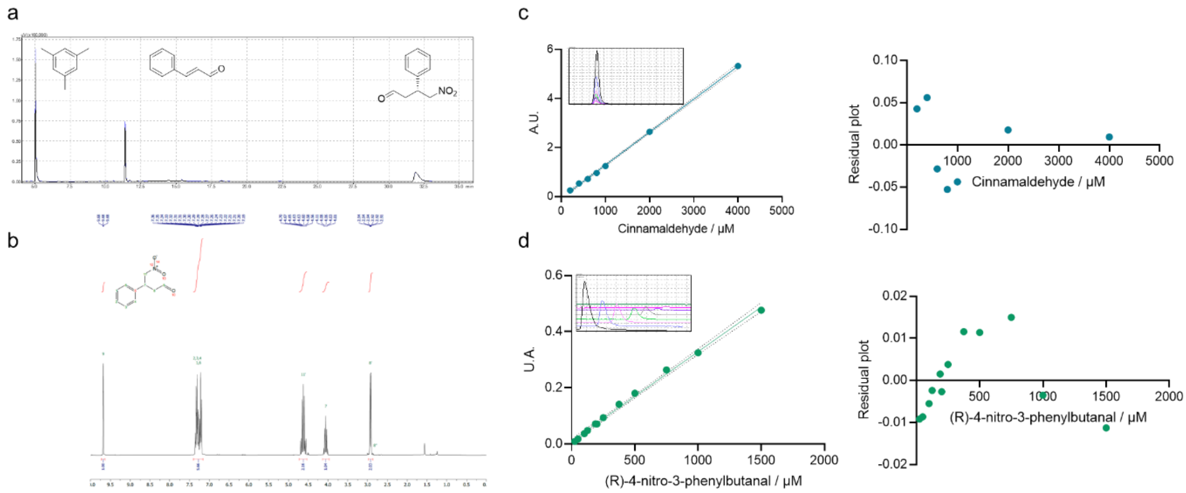

Figure S17. Analytics of substrate and product. Synthesis of 4-nitro-3-phenylbutanal was performed using 5 µM DERA-MA enzyme as a biocatalyst in a 100 mL small-scale reaction. The reaction was formulated with 5 mM *E*-cinnamaldehyde, 50 mM nitromethane, in 50 mM HEPES, 150 mM NaCl, and 10% ethanol, pH 6.5. The product was obtained after flash chromatography with a 50% yield. The sample was analysed by a) GC and b) NMR. Calibration curves were used to calculate substrate depletion and *E*-cinnamaldehyde in the product sample. c) A calibration curve using commercial (Merck) *E*-cinnamaldehyde was used to calculate the remnant substrate obtained in the biosynthesis, then d) the calibration curve of the product was recalculated accordingly. Chromatograms for each concentration of substrate and product, and the residual plots of the calibration curves are shown.

Please do not adjust margins
